# PATH-SURVEYOR: pathway level survival enquiry for immuno-oncology and drug repurposing

**DOI:** 10.1186/s12859-023-05393-y

**Published:** 2023-06-28

**Authors:** Alyssa N. Obermayer, Darwin Chang, Gabrielle Nobles, Mingxiang Teng, Aik-Choon Tan, Xuefeng Wang, Y. Ann Chen, Steven Eschrich, Paulo C. Rodriguez, G. Daniel Grass, Soheil Meshinchi, Ahmad Tarhini, Dung-tsa Chen, Timothy I. Shaw

**Affiliations:** 1 Department of Biostatistics and Bioinformatics, H. Lee Moffitt Cancer Center and Research Institute, Tampa, FL 33612 USA; 2 Department of Immunology, H. Lee Moffitt Cancer Center and Research Institute, Tampa, FL 33612 USA; 3 Morsani College of Medicine, University of South Florida, Tampa, FL 33612 USA; 4 Department of Oncological Sciences, Huntsman Cancer Institute, University of Utah, Salt Lake City, UT 84112 USA; 5 Department of Radiation Oncology, H. Lee Moffitt Cancer Center and Research Institute, Tampa, FL 33612 USA; 6 Clinical Research Division, Fred Hutchinson Cancer Research Center, Seattle, WA USA; 7 Children’s Oncology Group, Monrovia, CA USA; 8 Department of Cutaneous Oncology, H Lee Moffitt Cancer Center and Research Institute, Tampa, FL USA

**Keywords:** Pathway-level survival analysis, R Shiny app, Hazard ratio GSEA, Pathway clustering

## Abstract

Pathway-level survival analysis offers the opportunity to examine molecular pathways and immune signatures that influence patient outcomes. However, available survival analysis algorithms are limited in pathway-level function and lack a streamlined analytical process. Here we present a comprehensive pathway-level survival analysis suite, PATH-SURVEYOR, which includes a Shiny user interface with extensive features for systematic exploration of pathways and covariates in a Cox proportional-hazard model. Moreover, our framework offers an integrative strategy for performing Hazard Ratio ranked Gene Set Enrichment Analysis and pathway clustering. As an example, we applied our tool in a combined cohort of melanoma patients treated with checkpoint inhibition (ICI) and identified several immune populations and biomarkers predictive of ICI efficacy. We also analyzed gene expression data of pediatric acute myeloid leukemia (AML) and performed an inverse association of drug targets with the patient’s clinical endpoint. Our analysis derived several drug targets in high-risk KMT2A-fusion-positive patients, which were then validated in AML cell lines in the Genomics of Drug Sensitivity database. Altogether, the tool offers a comprehensive suite for pathway-level survival analysis and a user interface for exploring drug targets, molecular features, and immune populations at different resolutions.

**Supplementary Information:**

The online version contains supplementary material available at 10.1186/s12859-023-05393-y.

## Background

Organizing biological knowledge into pathways facilitates the integrative analysis of gene expression data derived from RNA sequencing and proteomics profiling. Common pathway-level analysis tools, such as ENRICHR [1] and GSEA [2], are able to perform pathway enrichment analysis based on gene set databases (e.g., KEGG [3], REACTOME [4], MSIGDB [5], LINCS1000 [6], and the Cell Marker database [7]). While these pathway analysis tools tend to focus on differentially expressed genes between two groups of samples, an alternative approach is to infer a pathway activity score in a single sample by transforming the expression of a set of genes into a single value using gene summary statistics, such as maxmean statistics [8], PLAGE [9], GSVA [10], and ssGSEA [2, 11]. These summary scores can capture pathway and gene regulatory activities in a sample, which is often challenging to infer by a single-gene expression. Furthermore, scores derived from custom gene sets or from network analyses [12–14] can then be used to dichotomize the patient population for survival analysis. For example, PERK-associated gene activity was found to be associated with a higher risk in melanoma patients [15], RAS dependency index was developed in pancreatic adenocarcinoma [16], LCK network activity was associated with T-cell acute lymphoblastic leukemia patient survival [17], and an epithelial-mesenchymal transition score was found to be associated with poorer disease-free survival in ovarian and colorectal patients [18]. Moreover, single scores derived from immune markers can be used as an estimate of immune components (e.g., Xcell [19], TIMER 2.0 [20], and geometric mean estimation of tumor infiltrative leukocytes [21]). These immune scores can then be applied in cancer patient classification [22], as a biomarker of checkpoint immunotherapy response [23], or as a prognosis marker that’s predictive of clinical outcome [24].

With several drug screening databases now available with perturbed gene sets after drug treatment in cancer cell lines, survival analysis can also be utilized to perform drug screening by identifying drugs that can reverse expression associated with highly refractory diseases [25]. For example, the Phase III AAML1031 clinical trial in pediatric acute myeloid leukemia (AML) has failed to show the benefit of experimental agents [26]. While preclinical studies have supported bortezomib as a therapeutic target in myeloid leukemias[27], bortezomib with standard chemotherapy did not improve treatment outcomes in children [26], which highlights a critical need for new therapeutic strategies in these pediatric AML patients. Thus, by leveraging pathway-level survival analysis, we can systematically screen for drug-induced targets that can reverse gene expression associated with high-risk cancer types, such as pediatric AML. Overall, these integrative summary scores represent a useful approach in highlighting signaling pathways, drug-induced targets, and immune populations that correlate with the clinical outcome. But existing survival analysis tools either lack a user-friendly interface or have limited functionality for systematic screening of large pathway databases. They are often restricted in available patient cohorts or limited to a small subset of pathways [28–30] and are often incapable of accepting external user input data [28–31] or clinically relevant covariates [29, 32, 33]. Thus, to facilitate the public mining of retrospective clinical studies, we introduce PATH-SURVEYOR, a comprehensive plug-and-play suite for pathway-level survival analysis of signature databases. Our tool is presented with the following unique features:

1. A one-stop tool for expression-based survival analyses.
2. The ability to include multiple covariates inside the Cox-proportion hazard pathway model.
3. The ability to summarize prioritized gene signatures into relevant clusters and pathway modules.
4. The ability to perform hazard ratio ranked gene set enrichment analysis.

Altogether, survival analysis is a critical branch of statistics for analyzing the time-to-event, and our tool facilitates a comprehensive survival analysis of pathway-level scores.

## Implementation

### Overview of the entire pipeline

**Fig. 1:**
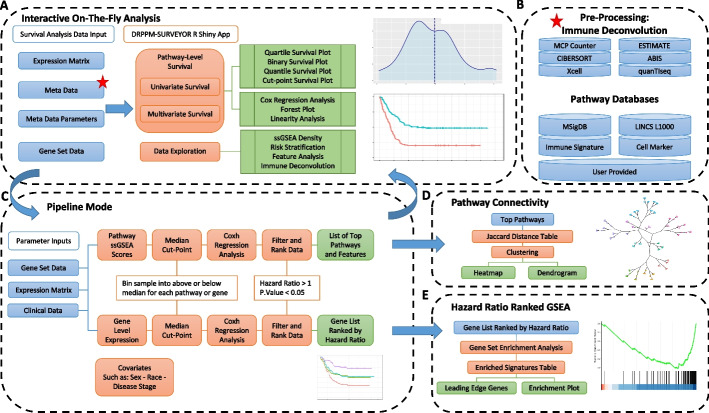
Schematic workflow of PATH-SURVEYOR: Pathway level survival enquiry for immune-oncology and drug repurposing. **A** In the On-The-Fly Shiny interface, users can provide a gene expression matrix matched with patient outcome meta information and a custom gene-set pathway. The shiny App will calculate a score for the selected pathway using a summary statistics, such as single sample gene set enrichment (ssGSEA), and dichotomized based on the median, optimum cut-point, quartiles, or user-specified cut-point. Univariate and multivariate Cox hazard regression analysis can be performed. **B** Several pathway databases are preloaded inside our app, including MsigDB, LINCS1000, and CellMarksDB. Additional patient features or immune deconvoluted features (Red Star Top Right) can be incorporated into a multivariate Cox hazard model and explored by our interface. **C** In the Pipeline mode, these scores were further dichotomized above and below the median ssGSEA score. A Cox regression analysis can be performed on each gene set to generate a comprehensive table of gene sets to filter according to significant, high-risk patients (hazard ratio > 1, *P* value < 0.05). A hazard ratio ranked gene list can be analyzed by GSEA (Bottom Right), and prioritized pathways can be visualized by pathway connectivity quantified by the Jaccard Index (Middle Right). Output of the analysis can be either a list of top pathways or gene list ranked based on hazard ratio. **D** Prioritized pathways can be visualized by pathway connectivity quantified by the Jaccard Index. **E** A hazard ratio ranked gene list can be analyzed by GSEA

PATH-SURVEYOR is implemented in the R environment, and packages can be automatically installed during runtime. There are four major components of the PATH-SURVEYOR (Fig. 1), which include: (1) The Interactive (UI) Mode (Fig. 1A). This feature allows for a point-and-click pathway survival analysis. The program can dichotomize the patient population based on gene expression (or pathway activity) into high and low levels by either median or a user-specified cutoff. The survival curve is estimated between patient populations using the Kaplan–Meier method, and differences in survival times are tested using Cox's proportional hazard. The interactive mode offers the ability to perform select immune deconvolution in real time and perform univariate or complex multivariate analyses of clinical features. (2) The Pipeline (Advanced) Mode (Fig. 1C). This feature performs a complete survival analysis of pathway databases and gene features. Covariates can be included as part of the systematic screening, and the P-values are corrected by Benjamini-Hochberg. (3) Pathway Connectivity. This feature allows the user to evaluate the similarity between pathways and group pathways that are predictive of the clinical outcome (Fig. 1D). (4) Hazard Ratio Ranked Gene Set Enrichment Analysis (GSEA) (Fig. 1E). This user interface performs a GSEA analysis based on the gene-level hazard ratio ranking derived from the Pipeline Mode. This feature facilitates the identification of clinically relevant pathways and, in turn, identifies regulators that can drive the underlying gene expression**.** Additional installation and usage instructions is available in the Additional file 9: Methods section.

### “On-the-fly” mode: shiny interface

PATH-SURVEYOR interactive mode provides the ability to analyze and visualize “on-the-fly” associations of immune signatures and pathways scores with a clinical endpoint (Fig. 1A). The application facilitates the partitioning of patients based on pathway scores, estimated immune cells, and gene expression level, followed by univariate Cox-regression survival analysis and multivariate Cox-regression analysis. PATH-SURVEYOR uses several R packages, including survival (v3.2–11), survminer (v0.4.9), GSVA (v1.40.1) [10], and immunedeconv [34] (v2.1.0). Preloaded pathways include gene sets from MsigDB [5], CellMarker [7], and LINCS1000 [6] (Fig. 1B). Pathway score is calculated with the gsva() function based on ssGSEA, GSVA, plage, or zscore. The ssGSEA score is used as the default method because its calculation is not dependent on other samples in the cohort. Immune deconvolution is performed with the immunedeconv R package, which includes several deconvolution packages, such as CIBERSORT [35], ESTIMATE [36], and MCP counter [37]. Based on the derived feature, patients are divided into high and low levels based on this pathway activity (or individual gene expression), using either the median, quartiles, or a cutoff specified by the user. For multivariate analysis, a covariate can be selected from the user-provided meta-information file. The multivariate survival analysis can be performed through additive and multiplicative interaction of two or more variables. To evaluate the association between pathways and survival over time is defined through a Cox-regression function [38]. Our tool allows for the pathway association with survival after adjusting for patient meta information and evaluates associated interactions between the pathway and patient meta information. Our tool also allows the user to assess the linearity of each covariate and the proportional hazard assumption.

### Pipeline mode: systematic pathway-level survival analysis

To facilitate the identification of top high-risk pathways and genes, we have developed a pipeline to systematically assess pathways associated with hazard by a Cox proportional hazard function (Fig. 1C). The user can provide or select individual genes and pathway databases to perform a systematic screening. Each expression feature is stratified based on a median cutoff. The user also has the option of performing a systematic screening with the inclusion of a covariate as an additive or multiplicative interactive model. The *P* value is calculated on the likelihood ratio, wald test. An adjusted P value can be calculated based on Benjamini–Hochberg correction method. In the output table, genes and pathways are ranked by the likelihood ratio *P* value.

### Connectivity Mode: Pathway Gene-set Connectivity

The Connectivity Mode offers the user the ability to analyze the similarity between pathways associated with survival (Fig. 1D). The hazard ratio ranked pathways from the pipeline mode can be used as input to the Pathway Connectivity R Shiny app to generate hierarchical clustering based on gene-set similarity. A Jaccard function can calculate the distance between pathways:

The Jaccard score function J for two pathways A and B is defined as

$${\text{J}}\left( {{\text{A}},{\text{ B}}} \right) = \left| {{\text{A}} \cap {\text{B}}} \right|/\left| {{\text{A}} \cup {\text{B}}} \right|$$

where, A contains n set of genes, A = [a1, a2, …, an], B contains m set of genes, B = [b1, b2, …, bm]

The Jaccard matrix can be visualized as a heatmap.

Next, the pathways can be clustered using the hclust function from R (v4.1.0) into k-groups (user-specified). Clusters can be visualized as a dendrogram. To overlap survival-associated gene expression, genes within the pathway can be displayed as a table with a flexible sorting feature and added annotation information.

### GSEA mode: hazard ratio ranked gene set enrichment analysis

From the pipeline mode, we can derive a hazard ratio ranked gene list, which can then be applied as input to the Pre-Ranked-Hazard-Ratio GSEA R Shiny app (Fig. 1E). This application takes a two-column file of the gene symbols and hazards ratios, which is used as input to the GSEA function from clusterProfiler (v4.0.5). The application performs GSEA, and results can be visualized as a table with additional options for visualizing the GSEA plots through the gseaplot2 function from enrichplot (v1.12.3). When screening a large number of pathways, the hazard-ratio ranked GSEA function would be more suitable due to its ability to perform rapid screening of gene set pathway databases compared to ssGSEA survival analysis (Additional file 8: Table S1). Moreover, Pre-Ranked-Hazard-Ratio GSEA would be more sensitive in capturing subtle differences in survival when analyzing pathways with a high number of genes, which is an inherent design of the GSEA algorithm [39]. The ssGSEA survival analysis would be more favorable when analyzing pathways with a limited number of genes, such as cell-type-specific markers. Overall, both strategies offer complementary support for a pathway to be associated with survival.

## Results

To demonstrate the functionalities of the PATH-SURVEYOR framework, we have included use-case examples of biomarker discovery in a cohort of immunotherapy-treated melanoma patients. We have also provided an example use-case strategy for drug repurposing in pediatric acute myeloid leukemia patients.

### Identifying immune pathways associated with effective checkpoint inhibition treatment

**Fig. 2:**
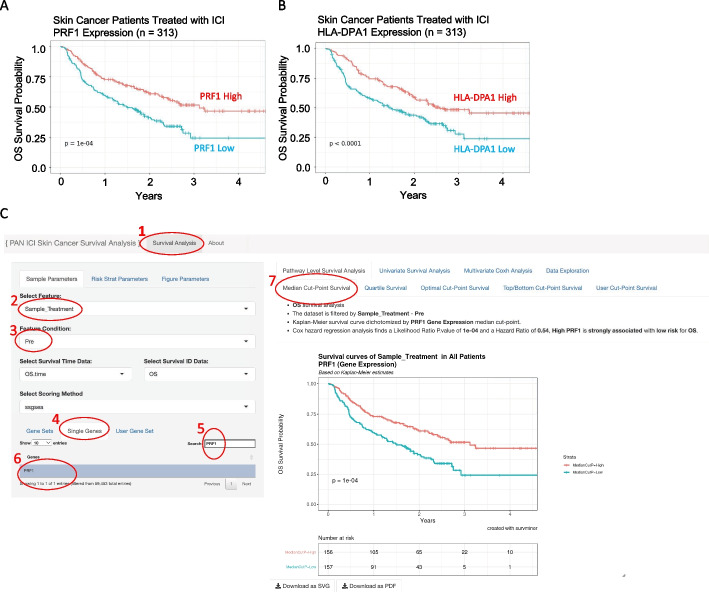
Overall survival curves of immunotherapy treated skin cancer patients. Overall survival curves of immunotherapy treated skin cancer patients dichotomized based on perforin 1 (PRF1) (**A**) and HLA-DPA1 (**B**). User interface of the gene-level survival analysis of PRF1. Samples were filtered based on pre-treatment (2 and 3). PF1 was searched and selected from the Single-gene list (4, 5, 6). Kaplan meier curve is shown comparing a median dichotemized patient population (7)

**Fig. 3:**
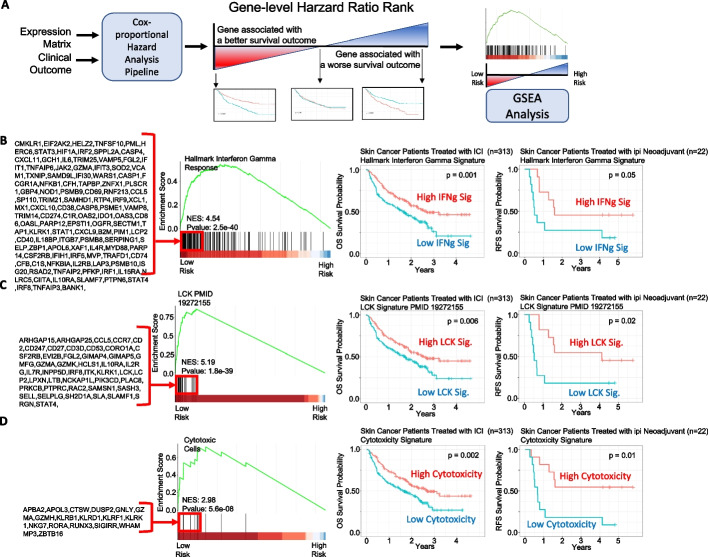
Gene-set Enrichment Analysis with Genes Ranked by Hazard Ratio. **A** Genes can be ranked based on the hazard ratio for overall survival (OS). GSEA can be applied to examine for similarity in gene features that are shared in high or low-risk diseases. Interferon Gamma (**B**), the LCK Signature (**C**), and the Cytotoxic signature (**D**) were found to be associated with low-risk patients based on GSEA. Kaplan Meier curves showing patients dichotomized based on ssGSEA immune signatures are shown on the right in skin cancer patients treated with ICI and validated in a separate cohort of patients treated with ipi adjuvant therapy

**Fig. 4:**
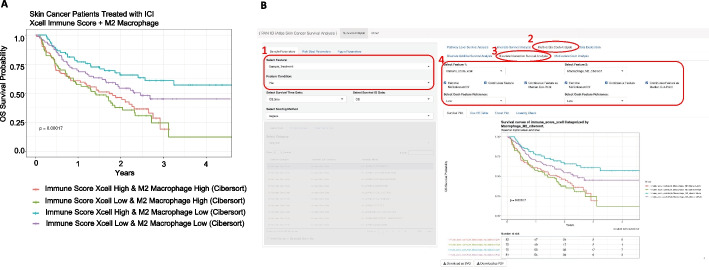
Associating Immune Scores with Patient Survival. **A** Skin Cancer Patients treated with ICI dichotemized based on xCell derived Immune Scores as well as M2 Macrophage estimated from Cibersort. Patients with High Immune Score and Low M2 Macrophage were associated with better survival**. B** User Interface that access this function from the Shiny App is (1) Select Feature Condition to restrict to pretreated patients, (2) Multivariate Coxh Analysis, (3) Bivariate Interaction Survival Analysis, (4) Select Immune Score (on left) and M2 Macrophage (on right). The feature can be dichotemized “on-the-fly” if it is a continuous variable (4)

**Fig. 5:**
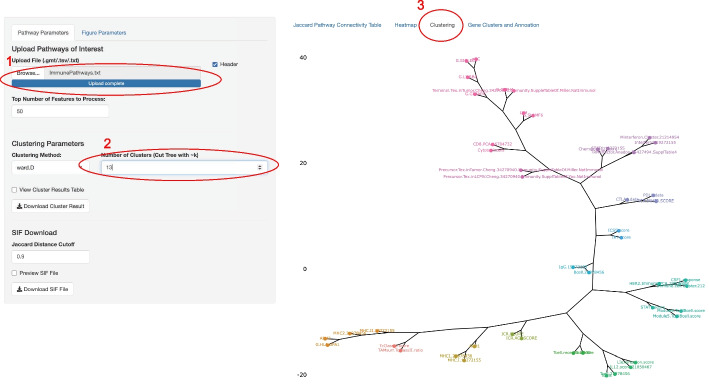
Pathway connectivity Analysis User Interface. (1) Input list of pathway that satisfy the selection criterion. (2) Set the number of clusters captured by the algorithm. (3) Select the Clustering Visualization Tab

To identify predictive biomarkers that facilitate patient selection of patients suitable for immune checkpoint inhibitor (ICI) treatment, we integrated 313 melanoma patients treated with ICI from Riaz et al. (n = 51) [40], Hugo et al. (n = 25) [41], Van Allen et al. (n = 25) [42] (n = 42), Liu et al. (n = 122) [43], and Gide et al. (n = 73) [44]. First, we performed a systematic univariate Cox-hazard analysis of individual gene expression in the “Pipeline Mode” and identified 100 genes associated with the better prognosis (Additional file 8: Table S2). These include PRF1 and HLA-DPA1, which have been previously reported as predictive biomarkers for ICI therapy [45] (Fig. 2). “On-the-fly analysis mode” further demonstrate PRF1 and HLA-DPA1 had significantly higher expression in patients who respond to ICI treatment (Additional file 1: Figure S1). We then ranked the genes based on hazard ratio derived from the Cox-proportion hazard model (Additional file 2: Figure S2A) and performed a Hazard Ratio Ranked GSEA analysis of the Hallmark database (Fig. 3A; Additional file 2: Figure S2B, C). Interferon Gamma was found associated with Low-risk patients (Fig. 3B). Consistently, immune signatures associated with LCK and Cytotoxicity were also associated with Low-risk patients (Fig. 3C, D). Significantly, these immune signatures were also validated in 22 patients treated with neoadjuvant ipilimumab [46] (Fig. 3B–D) and in 88 patients from a Moffitt Melanoma Cohort with metastatic disease [46] (Additional file 3: Figure S3). Through immune deconvolution, we derived an immune score from xCell [19] and an estimated M2-like macrophage population from Cibersort [35]. We found that high immune infiltration with low M2-like (immune suppressive) macrophages was associated with a better outcome (Fig. 4A, B). Next, we used the “Pipeline Mode” to perform a systematic univariate Cox-hazard analysis of gene expression followed by a GSEA analysis of immune signatures and identified to identify 69 immune signatures associated with a better outcome (Additional file 8: Table S3). A systematic assessment of the immune pathway followed by Pathway Connectivity analysis demonstrated 13 immune modules captured a favorable outcome in pretreated RNA samples, including CD8 cytotoxicity, antigen presentation, interferon, and immune checkpoint marker signatures (Fig. 5, Additional file 4: Figure S4). Next, we used the MsigDB hallmark pathways to compare performance between the hazard-ratio ranked GSEA analysis and ssGSEA analysis. We find that 72% of the (8/11) hallmark pathways identified by ssGSEA can be identified by the hazard-ratio Ranked GSEA approach. The eight overlapping pathways were consistently associated with an active immune response with a favorable prognosis (Additional file 5: Figure S5). The hazard-ratio Ranked GSEA was also able to correlate high-risk patients with nine pathways associated with MYC and glycolysis, which was not identified by the ssGSEA survival analysis (Additional file 5 Figure S5). Altogether, our tool suggests favorable outcome is linked with immune activation while highlighting the possibility of MYC-driven immune suppression in high-risk melanoma patients.

### Survival-directed therapeutic discovery in acute myeloid leukemia

**Fig. 6:**
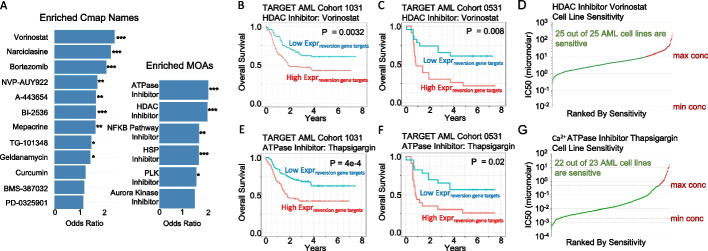
Overall survival curves of KMT2A positive patients in TARGET AML 1031 and AML 0531. **A** Odds ratio of the top enriched Cmap names and mechanisms of action (MOAs) identified through EFS Coxph regression analysis. **B** Overall survival curves of KMT2A fusion positive patients in TARGET AML 1031 (N = 220). Patients are classified by the ssGSEA score derived from genes affected by the HDAC inhibitor Vorinostat. **C** Overall survival curves of KMT2A fusion positive patients in TARGET AML 0531 (N = 46) validation cohort. Patients are classified by the ssGSEA score derived from genes affected by the HDAC inhibitor Vorinostat. **D** Cell-line sensitivity ranking based on IC50 values of Vorinostat. **E** Overall survival curves of KMT2A positive patients in TARGET AML 1031. Patients are classified by the ssGSEA score derived from genes affected by the ATPase inhibitor Thapsigargin. **F** Overall survival curves of KMT2A positive patients in TARGET AML 0531 validation cohort. Patients are classified by the ssGSEA score derived from genes affected by the ATPase inhibitor Thapsigargin. **G** Cell-line sensitivity ranking based on IC50 values of Thapsigargin

To leverage our framework for therapeutic discovery, we obtained the gene expression data and clinical annotation of 220 patients with the KMT2A fusion event from the National Cancer Institute TARGET pediatric acute myeloid leukemia (AML) 1031 cohort (0–22 years of age). The translocation event of the gene KMT2A, also known as mixed lineage leukemia (MLL), is frequently identified in pediatric AML. Through its multiple fusion partners arises a diverse patient population with a need for advanced risk stratification [43]. Through the PATH-SURVEYOR suite of tools, we examined pathways and genes associated with poor outcomes and idenfied therapeutic targets in high-risk patients. First, single samples gene set enrichment analysis (ssGSEA) was performed using the expression data in tandem with the Library of Integrated Network-based Cellular Signatures (LINCS; 31,028 gene sets) LINCS1000 gene sets to calculate the pathway scores (Additional file 6: Figure S6). Next, the patients were dichotomized through a median cut-point of each pathway score into an above-median or below-median group, followed by a Cox proportional hazards regression using the patient’s overall survival (OS). A hazard ratio value greater than one and a P-value less than 0.05 was applied to identify significant pathways associated with high risk. To prioritize a putative therapeutic target that downregulates genes associated with high-risk AML in the KMT2A subgroup, we examined the enriched connectivity map (cMAP) name and Mechanism of Action. We found 12 enriched Cmap names and 6 enriched drug categories grouped by their mechanism of action (Fig. 6A). Notably, genes downregulated by the HDAC inhibitor, Vorinostat, and ATPase inhibitor, Thapsigargin, were associated with the worst prognosis based on OS and event-free survival (EFS) (Fig. 6B, E). The two prioritized targets were also validated in patients after resampling 75% of the population (Additional file 7: Figure S7) and were further validated in a separate cohort of pediatric AML patients from AAML0531 (#NCT00372593) (Fig. 6C, F). The proportional hazard regression models were evaluated to be suitable based on a Chi-square Goodness-of-Fit test (Additional file 8: Table S4). Furthermore, both Vorinostat and Thapsigargin were highly sensitive in AML cell lines based on the genomics of drug sensitivity in cancer (GDSC) database (Fig. 6D, G). Taken together, we presented an integrative strategy utilizing PATH-SURVEYOR to prioritize pathways based on patient risk and identified known therapeutic targets in high-risk KMT2A fusion-positive AML patients.

## Discussion

PATH-SURVEYOR is designed to visualize and perform systematic survival analysis based on gene and pathway information. The application is designed for users with limited experience in programming as well as for advanced users to perform systematic high-throughput pathway screening. In the interactive mode, the Shiny application will ensure reproducibility and can be easily set up and applied in any cohort. In the pipeline mode, the user can apply univariate and multivariate analysis of pathway and patient covariates associated with patient survival outcomes. Our current application can also perform GSEA based on hazard ratio ranking as well as pathway clustering to examine shared gene and pathway features associated with survival, which is unique to PATH-SURVEYOR when compared with other tools, including TRGAted [31], UALCAN [28], Path2Surv [47], METABRIC-pathway-survival [48], CASA [32], GNOSIS[49], Esurv [33], Kmplot [29], and Survial Genie [30] (Table 1). Moreover, our tool offers the unique ability to perform quality assessment of the model and covariates, such as evaluation of the Cox hazard model assumption and linearity of each covariate. The PATH-SURVEYOR framework analyzed data from melanoma patient samples collected prior to ICI treatment, and we found cytolytic T cell and antigen presentation signatures were associated with a better survival outcome. Our result highlighted the potential of leveraging immune biomarkers as predictors of immunotherapy response in melanoma patients. In our analysis of pediatric AML patient samples, our result highlighted Vorinostat and Thapsigargin as drugs with the potential to reverse gene expression associated with high-risk KMT2A-fusion-positive patients. Interestingly, AML cell lines were sensitive to these drugs based on results from the GDSC database, underlining epigenetic regulators and endoplasmic reticulum stress as therapeutic targets in high-risk KMT2Ar AML cells. Altogether, we have provided two major use-case examples of utilizing our PATH-SURVEYOR framework for identifying immune biomarkers and drug repurposing of targets.

**Table 1:** Feature comparisons

Features	TRGAted	UALCAN	Path2Surv	METABRIC-pathway-survival	GNOSIS	CASAS	Esurv	Kmplot	Survival Genie	PATH-SURVEYOR
Pathway-level Survival Analysis	No	No	Yes	Yes	No	No	Yes	No	Yes	Yes
Network Connectivity Analysis	No	No	No	No	No	No	No	No	No	Yes
Hazard ratio-ranked GSEA Pathway Analysis	No	No	No	No	No	No	No	No	No	Yes
Immune-deconvolution Survival Analysis	No	No	No	No	No	No	No	No	Yes	Yes
Gene-level Survival Analysis	No	**Yes**	No	No	Yes	No	Yes	Yes	Yes	Yes
Univariate Survival Analysis of Other Patient/Sample Features	No	No	Yes	Yes	Yes	Yes	No	No	Yes	Yes
Multivariate Survival Analysis	No	**Yes**	No	Yes	Yes	No	No	Yes	No	Yes
Examining Covariate Distributions and associations (QC)	No	**Yes**	No	No	Yes	No	No	No	No	Yes
Plug-and-Play User Inputs	No	No	No	No	No	Yes	Yes	Yes	No	Yes

## Conclusion

As more RNA sequencing and proteomics data are being captured in large clinical trials, generating user- interfaces will facilitate access to these data sets. Thus, we present PATH-SURVEYOR, a program for inferring survival through pathways, immune components, and drug-induced targets. We anticipate PATH-SURVEYOR will enable a collaborative environment for exploring pathway-level, drug targets, and immune features that are predictive of treatment efficacy, especially for high-risk malignancies.

## Availability and requirements

Project name: PATH-SURVEYOR. Project home page: https://github.com/shawlab-moffitt/PATH-SURVEYOR-Suite. URL Links to the Input File Prep App: https://shawlab-moffitt.shinyapps.io/path_surveyor_fileprep/. URL Links to the PATH-SURVEYOR App: https://shawlab-moffitt.shinyapps.io/path_surveyor/. Preloaded Examples: https://shawlab-moffitt.shinyapps.io/path_surveyor_preloaded_example_aml/. https://shawlab-moffitt.shinyapps.io/path_surveyor_preloaded_example_melanomaici/. URL Links to the Connectivity Analysis App: https://shawlab-moffitt.shinyapps.io/pathway_connectivity/. Preloaded Example: https://shawlab-moffitt.shinyapps.io/pathway_connectivity_preloaded_example_melanomaici/. URL Links to the Hazard Ratio GSEA App: https://shawlab-moffitt.shinyapps.io/preranked_hazardratio_gsea/. Preloaded examples: https://shawlab-moffitt.shinyapps.io/preranked_hazardratio_gsea_preloaded_example_melanomaici/. Data and Code to the Example Use Cases: http://shawlab.science/shiny/PATH_SURVEYOR_ExampleUseCases/. Github Repository of the Supplementary Examples: https://github.com/shawlab-moffitt/PATH-SURVEYOR_Manuscript_Supplementary. Downloadable Instructions for setting up the Docker Images are available here: https://github.com/shawlab-moffitt/PATH-SURVEYOR-Suite/tree/main/7-PATH-SURVEYOR-Docker. Operating system: Platform independent. Programming language: R version 4.1 or higher. License: BSD License.

## Supplementary Information

**Additional file 1**. Supplementary Figure S1.

**Additional file 2**. Supplementary Figure S2.

**Additional file 3**. Supplementary Figure S3.

**Additional file 4**. Supplementary Figure S4.

**Additional file 5**. Supplementary Figure S5.

**Additional file 6**. Supplementary Figure S6.

**Additional file 7**. Supplementary Figure S7.

**Additional file 8**. Supplementary Table S1–S4.

**Additional file 9**. Supplementary Method.

**Additional file 10**. PATH-SURVEYOR-Suite-main.zip.

## Data Availability

An example of the Shiny app with user upload function is available here https://shawlab-moffitt.shinyapps.io/path_surveyor/ with documentation here https://github.com/shawlab-moffitt/PATH-SURVEYOR-Suite/tree/main/6-PATH-SURVEYOR-UserInput-App. An overview of the PATH-SURVEYOR Suite of tools can be found on our GitHub page (https://github.com/shawlab-moffitt/PATH-SURVEYOR-Suite), which includes source code, example data, and an installation guide. An example startup page is available to guide through the PATH-SURVEYOR-Suite with downloadable example files and example scripts. The processed RNA sequencing data were downloaded from iATLAS https://www.cri-iatlas.org/. The raw RNA-seq data for the TARGET data can be downloaded from the Database of Genotypes and Phenotypes (dbGaP) under the study ID phs000465.v21.p8. Subject to the NIH Genomic Data Sharing Policy, the raw data are freely available to all researchers via https://www.ncbi.nlm.nih.gov/projects/gap/cgi-bin/study.cgi?study_id=phs000465.v21.p8. Processed data are available at the National Cancer Institute’s Genomic Data Commons (https://portal.gdc.cancer.gov/) under the TARGET-AML project.ee Supplementary Method for additional details on installation and requirements on file inputs.

## Abbreviations

AML: Acute Myeloid Leukemia
GSEA: Gene Set Enrichment Analysis
GDSC: Genomics of drug sensitivity in cancer database
ICI: Immune checkpoint inhibitor
Ipi: Ipilimumab
Neo: Neoadjuvant
OS: Overall survival
CMAP: Connectivity Map
MOA: Mechanism of Action
TARGET: Therapeutically Applicable Research To Generate Effective Treatments
