# Supplementary material for: PATH-SURVEYOR: pathway level survival enquiry for immuno-oncology and drug repurposing
Source: BMC Bioinformatics. 2023 Jun 28;24:266. doi: 10.1186/s12859-023-05393-y (PMC10303868; doi:10.1186/s12859-023-05393-y)
Supplement: Supplementary file 1 — Additional file 1. Supplementary Figure S1. [file 12859_2023_5393_MOESM1_ESM.pdf]

Supplementary Figure S1.

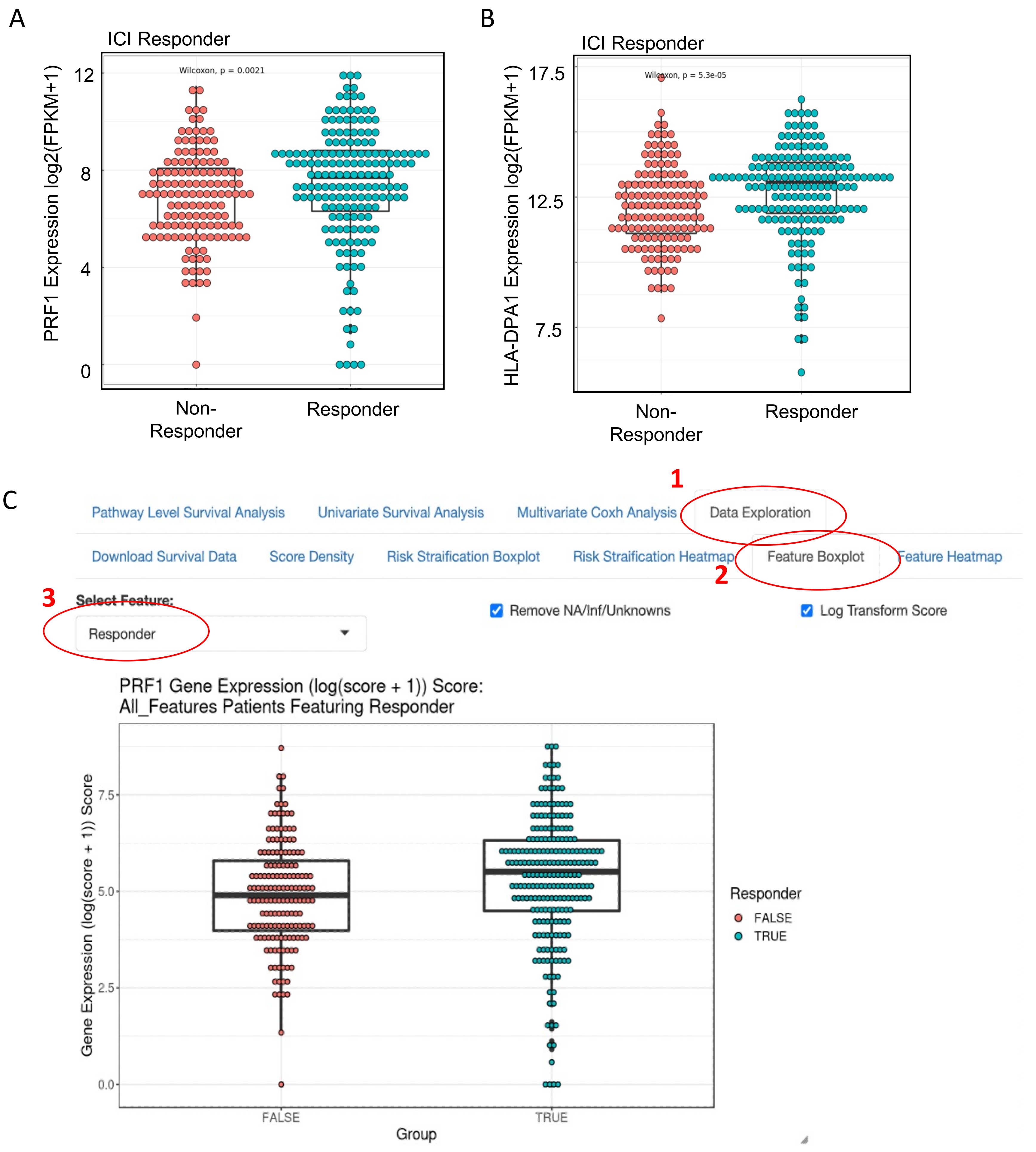

Supplementary Figure S1. Associating PRF1 (A) and HLA-DPA1 (B) expression with ICI Treatment Response in melanoma patients. C) User interface can be accessed from the Data Exploration tab (1) then from the Feature Boxplot tab (2) with the selected feature: “Responder” (3).
