# Supplementary material for: PATH-SURVEYOR: pathway level survival enquiry for immuno-oncology and drug repurposing
Source: BMC Bioinformatics. 2023 Jun 28;24:266. doi: 10.1186/s12859-023-05393-y (PMC10303868; doi:10.1186/s12859-023-05393-y)
Supplement: Supplementary file 2 — Additional file 2. Supplementary Figure S2. [file 12859_2023_5393_MOESM2_ESM.pdf]

Supplementary Figure S2.

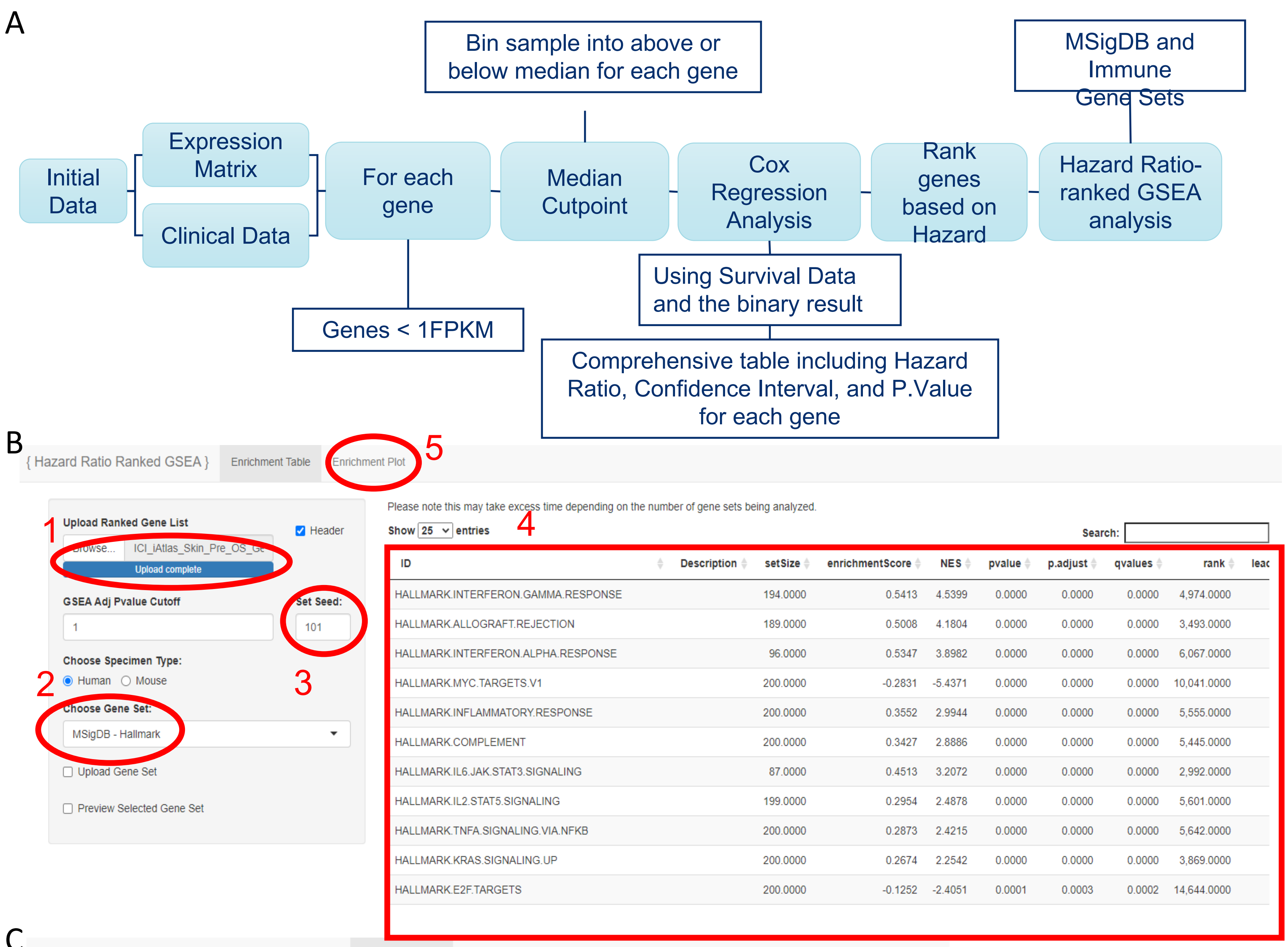

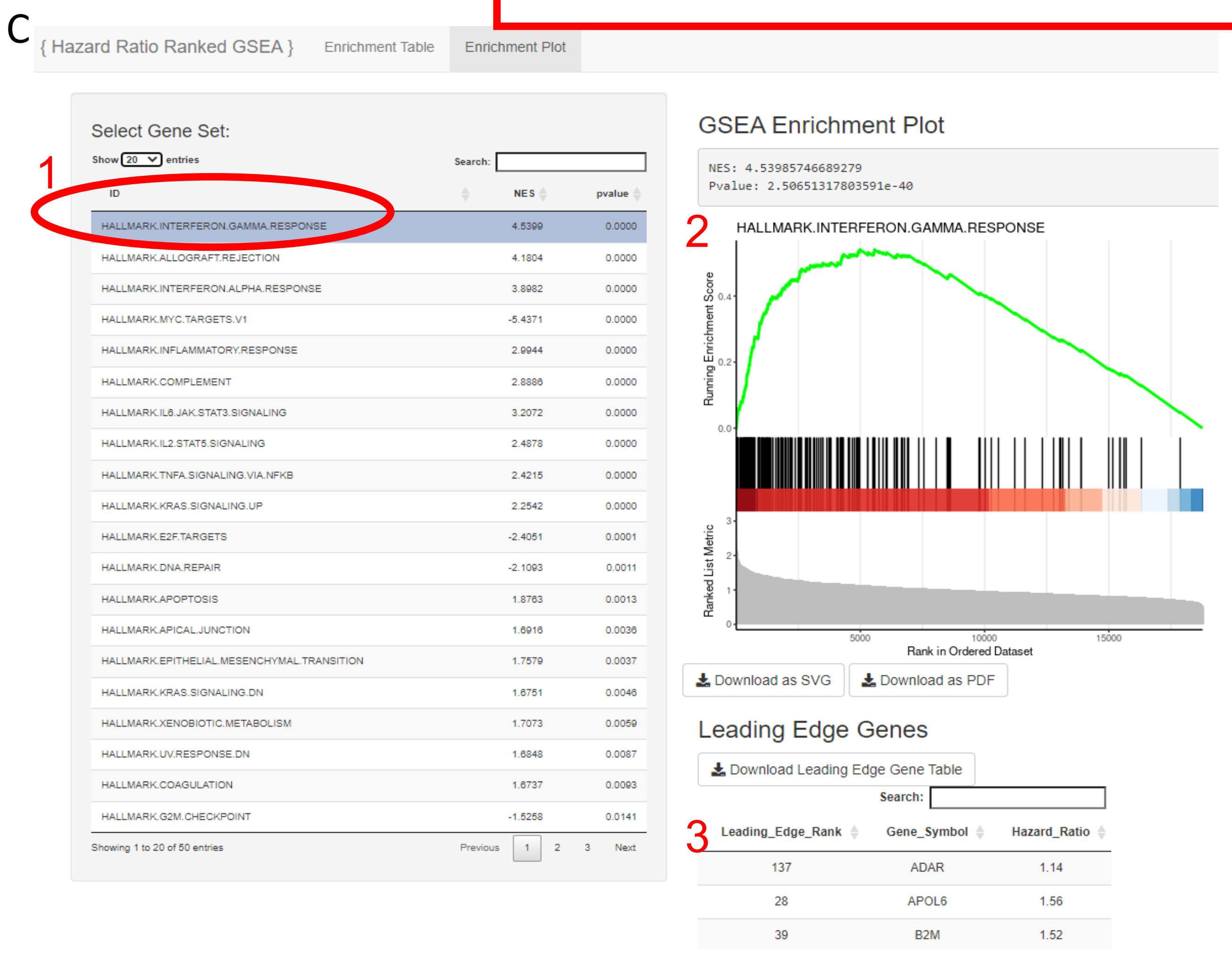

Supplementary Figure S2. A) Schematic workflow of gene-level survival analysis followed by hazard ratio gene set enrichment analysis. With the input data of an expression matrix, clinical data. Patients were binned into categories of above and below the median for each gene expression level. A cox regression analysis was performed on each gene expression to generate a comprehensive table of gene sets to filter according to significant, high-risk patients (hazard ratio > 1, p.value < 0.05). GSEA was then performed in MsigDB and Immune Gene Sets. B) Hazard Ratio Ranked GSEA User Interface: 1) The user must upload the output derived from pipeline mode of the Cox regression analysis pipeline. 2) Next select the gene set pathway database to use for performing the GSEA on their data. 3) A seed can be set to ensure reproducibility. 4) The page will load and then present the enriched signatures table. 5) The enriched pathways can be visualized in the "Enrichment Plot" tab. C) Hazard Ratio Ranked GSEA User Interface: 1) The user can select pathways from the enrichment table that was derived. 2) These pathways as well as their normalized enrichment score and Pvalue can be visualized and downloaded. 3) The user can view the leading-edge genes ranked from the pathway they are visualizing.
