# Supplementary material for: PATH-SURVEYOR: pathway level survival enquiry for immuno-oncology and drug repurposing
Source: BMC Bioinformatics. 2023 Jun 28;24:266. doi: 10.1186/s12859-023-05393-y (PMC10303868; doi:10.1186/s12859-023-05393-y)
Supplement: Supplementary file 3 — Additional file 3. Supplementary Figure S3. [file 12859_2023_5393_MOESM3_ESM.pdf]

Supplementary Figure S3.

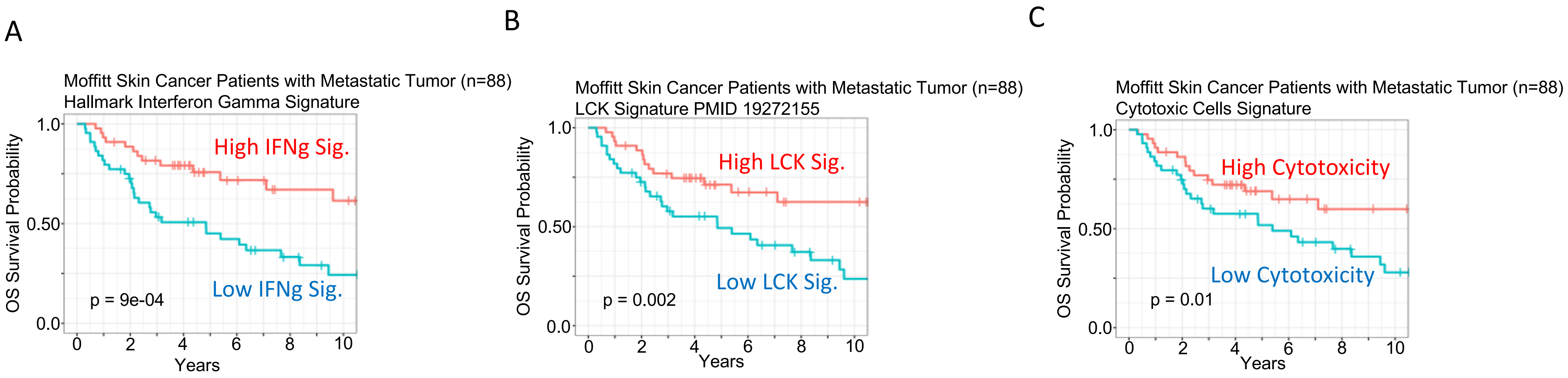

Supplementary Figure S3. Survival analysis in Moffitt Skin Cancer Patients with metastatic tumors. Interferon Gamma (A), the LCK Signature (B), and the Cytotoxic signature (C) were found to be associated with low-risk patients based on GSEA. Kaplan Meier curves showing patients dichotomized based on ssGSEA immune signatures are shown on the right.
