# Supplementary material for: PATH-SURVEYOR: pathway level survival enquiry for immuno-oncology and drug repurposing
Source: BMC Bioinformatics. 2023 Jun 28;24:266. doi: 10.1186/s12859-023-05393-y (PMC10303868; doi:10.1186/s12859-023-05393-y)
Supplement: Supplementary file 4 — Additional file 4. Supplementary Figure S4. [file 12859_2023_5393_MOESM4_ESM.pdf]

Supplementary Figure S4.

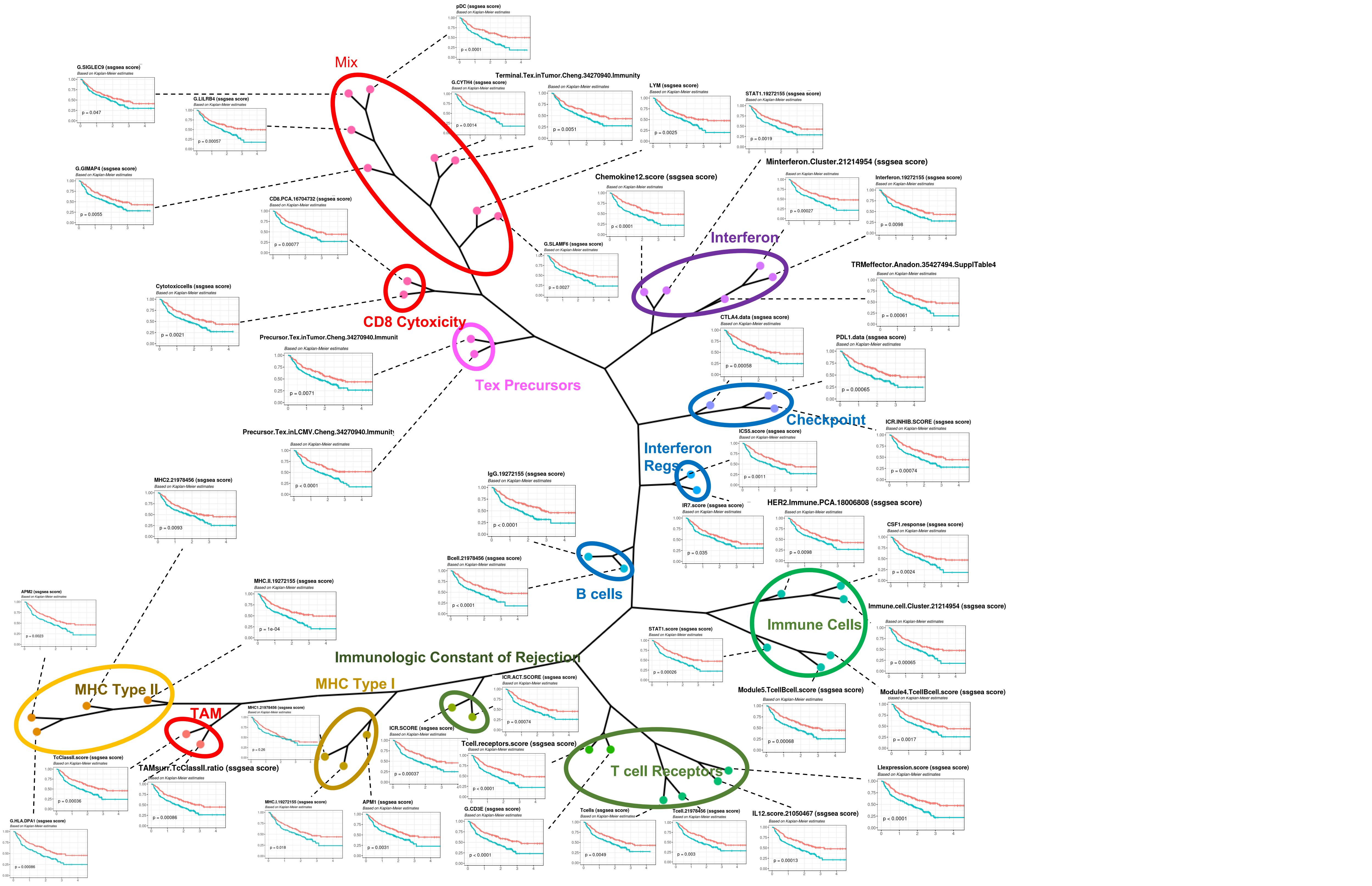

Supplementary Figure S4. 12 Immune Hubs assocaited with a favorable outcome in skin cancer patients treated with ICI. Pathways were clustered by a Jaccard distance calculated based on overlapping genes. Major immune categories were individually circled.
