# Supplementary material for: PATH-SURVEYOR: pathway level survival enquiry for immuno-oncology and drug repurposing
Source: BMC Bioinformatics. 2023 Jun 28;24:266. doi: 10.1186/s12859-023-05393-y (PMC10303868; doi:10.1186/s12859-023-05393-y)
Supplement: Supplementary file 5 — Additional file 5. Supplementary Figure S5. [file 12859_2023_5393_MOESM5_ESM.pdf]

Supplementary Figure S5.

A

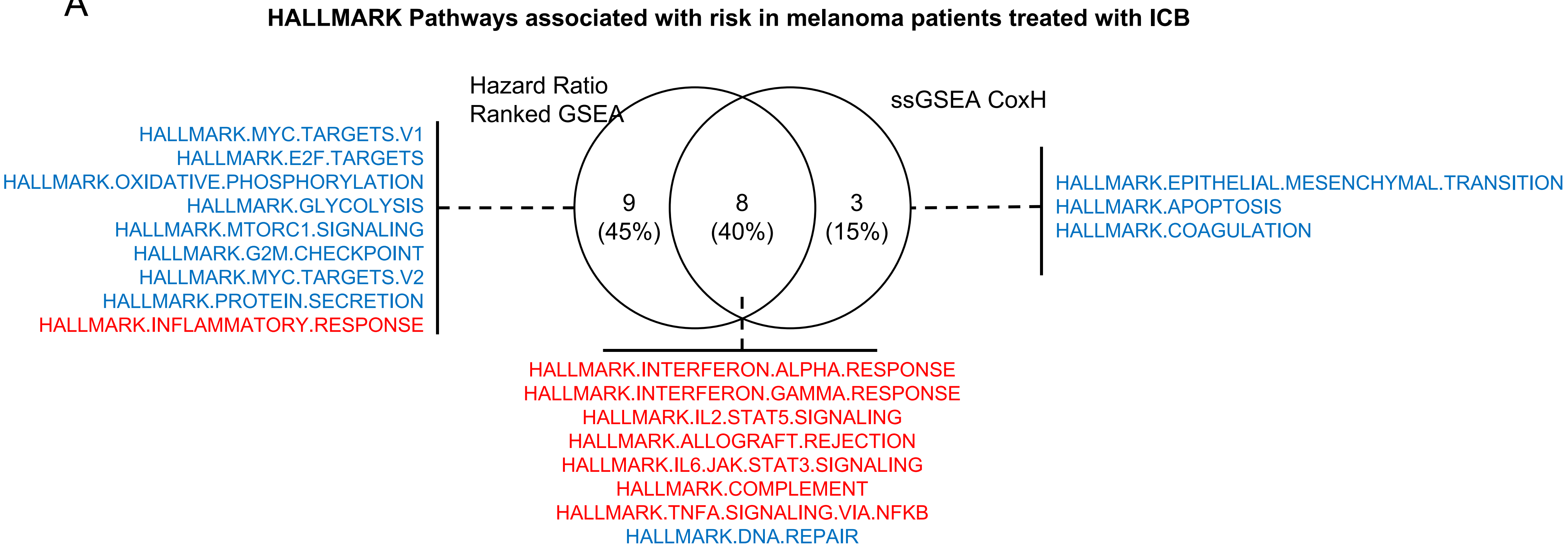

Supplementary Figure S5. Comparing the Hallmark pathways identified by hazard ratio ranked GSEA and ssGSEA survival analysis coxh associated with survival. Pathways consistently associated risk were derived based on p-value < 0.05 for both HR-ranked GSEA and ssGSEA CoxH approach. Pathways associated with the worst outcome are colored in blue. Pathways related to a better outcome are colored in red.
