# Supplementary material for: PATH-SURVEYOR: pathway level survival enquiry for immuno-oncology and drug repurposing
Source: BMC Bioinformatics. 2023 Jun 28;24:266. doi: 10.1186/s12859-023-05393-y (PMC10303868; doi:10.1186/s12859-023-05393-y)
Supplement: Supplementary file 6 — Additional file 6. Supplementary Figure S6. [file 12859_2023_5393_MOESM6_ESM.pdf]

Supplementary Figure S6.

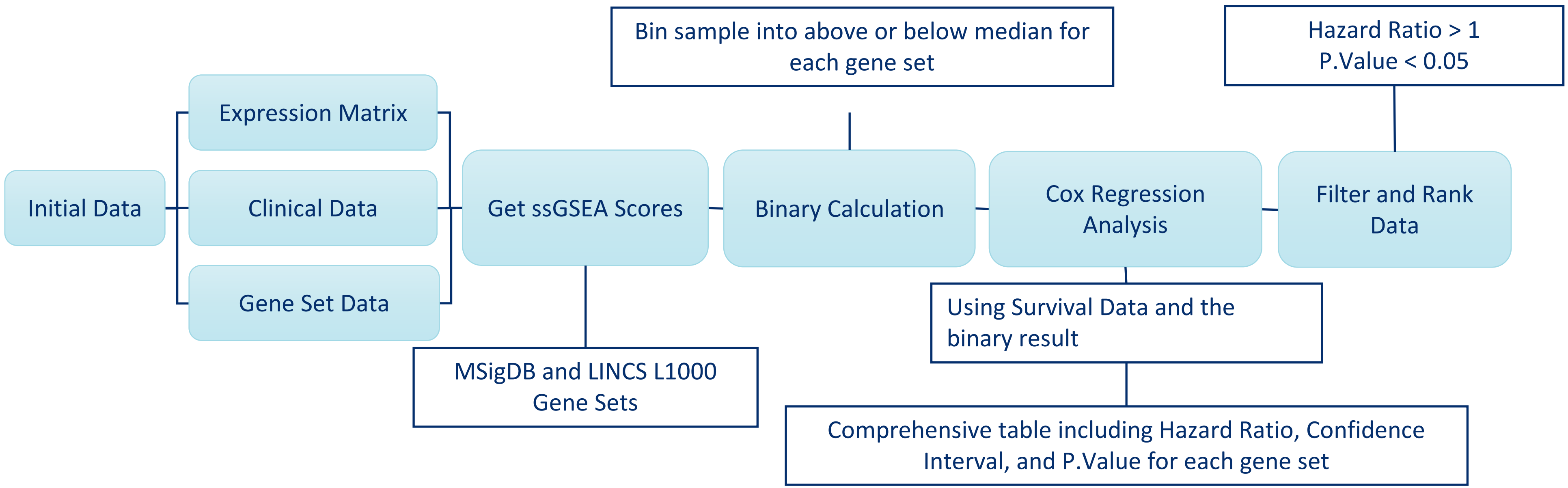

Supplementary Figure S6. Schematic workflow of ssGSEA Pathway Analysis method. With the input data of an expression matrix, clinical data, and a gene set file, single sample gene set enrichment (ssGSEA) scores were calculated. These scores were further binned into categories of above and below the median ssGSEA score for each gene set. A cox regression analysis was performed on each gene set to generate a comprehensive table of gene sets to filter according to significant, high-risk patients (hazard ratio > 1, p.value < 0.05).
