# Supplementary material for: PATH-SURVEYOR: pathway level survival enquiry for immuno-oncology and drug repurposing
Source: BMC Bioinformatics. 2023 Jun 28;24:266. doi: 10.1186/s12859-023-05393-y (PMC10303868; doi:10.1186/s12859-023-05393-y)
Supplement: Supplementary file 7 — Additional file 7. Supplementary Figure S7. [file 12859_2023_5393_MOESM7_ESM.pdf]

Supplementary Figure S7.

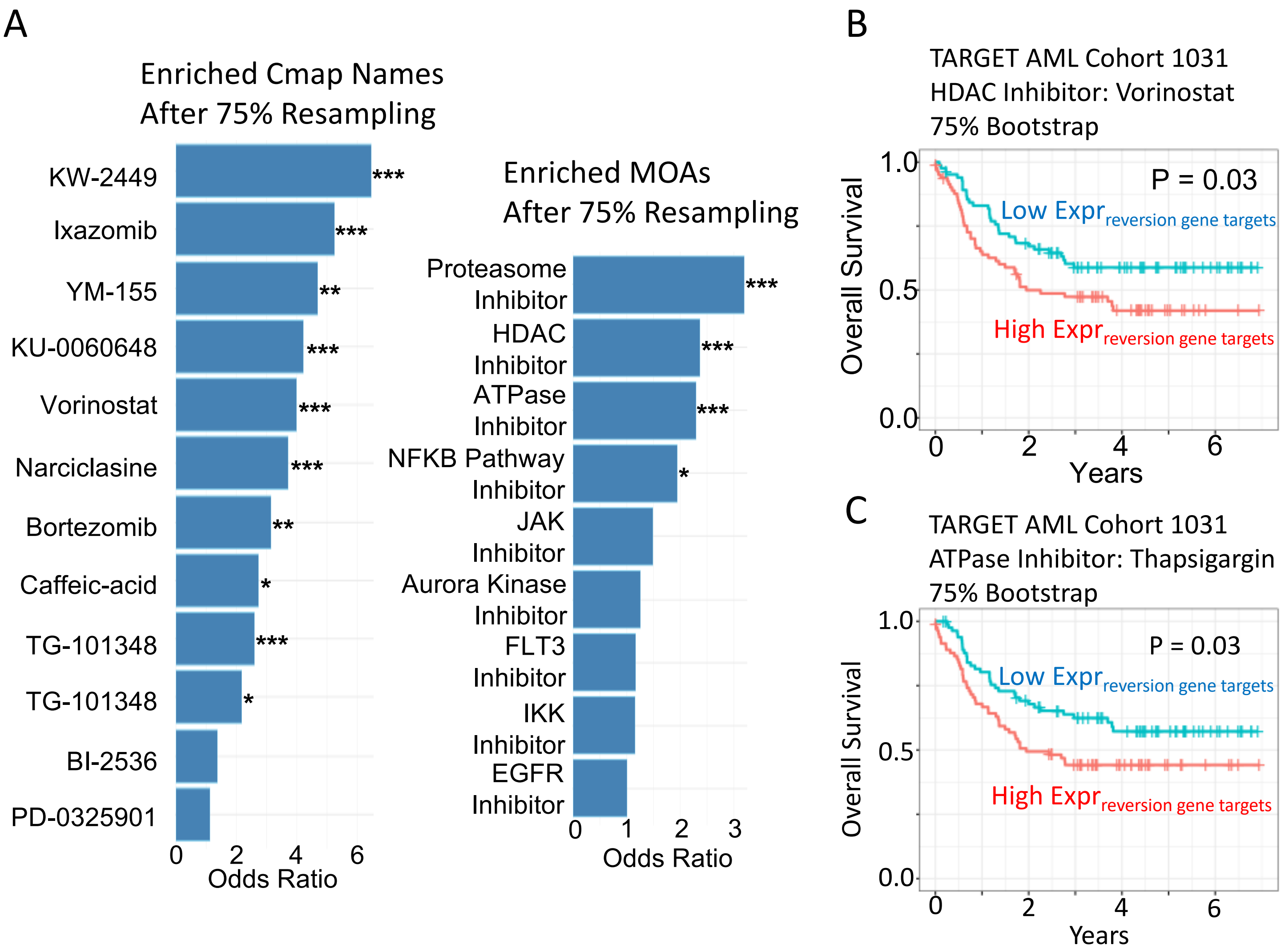

Supplementary Figure S7. Result after resampling 75% patients. A) Odds ratio of the top enriched Cmap names and MOAs identified through a 75% resampling of the OS Coxph regression analysis. B) Overall and event free survival curves of a 75% bootstrap of KMT2A fusion positive patients in TARGET AML 1031. Patients are classified by the ssGSEA score derived from genes affected by the HDAC inhibitor Vorinostat. C) Overall survival curves of a 75% resampling of KMT2A fusion positive patients in TARGET AML 1031. Patients are classified by the ssGSEA score derived from genes affected by the ATPase inhibitor Thapsigargin.
