# Supplementary material for: PATH-SURVEYOR: pathway level survival enquiry for immuno-oncology and drug repurposing
Source: BMC Bioinformatics. 2023 Jun 28;24:266. doi: 10.1186/s12859-023-05393-y (PMC10303868; doi:10.1186/s12859-023-05393-y)
Supplement: Supplementary file 9 — Additional file 9. Supplementary Method. [file 12859_2023_5393_MOESM9_ESM.docx]

**PATH-SURVEYOR**: **PATH**way Level **SURV**ival **E**nquir**Y** for Immuno-**O**ncology and Drug **R**epurposing.

Alyssa N. Obermayer^1^, Darwin Chang^2^, Gabrielle Nobles^3^, Mingxiang Teng^1^, Aik-Choon Tan^1,4^, Xuefeng Wang^1^, Steven Eschrich^1^, Y Ann Chen^1^, Paulo C. Rodriguez^2^, G. Daniel Grass^5^, Soheil Meshinchi^6,7^, Ahmad Tarhini^8^, Dung-tsa Chen^1^, Timothy I. Shaw^1*^

1. Department of Biostatistics and Bioinformatics. H. Lee Moffitt Cancer Center and Research Institute, Tampa, FL 33612, USA.
2. Department of Immunology, H. Lee Moffitt Cancer Center and Research Institute, Tampa, FL 33612, USA.
3. Morsani College of Medicine. University of South Florida. Tampa, FL 33612, USA.
4. Department of Oncological Sciences, Huntsman Cancer Institute, University of Utah, Salt Lake City, UT 84112, USA.
5. Department of Radiation Oncology. H. Lee Moffitt Cancer Center and Research Institute, Tampa, FL 33612, USA
6. Clinical Research Division, Fred Hutchinson Cancer Research Center, Seattle, WA
7. Children's Oncology Group, Monrovia, CA
8. Department of Cutaneous Oncology, H Lee Moffitt Cancer Center and Research Institute, Tampa, Florida, USA

* Corresponding

**Comprehensive Tutorial and Installation on GitHub**

An overview of the PATH-SURVEYOR Suite of tools can be found on our GitHub page ([https://github.com/shawlab-moffitt/PATH-SURVEYOR-Suite](https://github.com/shawlab-moffitt/DRPPM-PATH-SURVEIOR-Suite)), which includes source code, example data, and an installation guide. An example startup page is available to guide through the PATH-SURVEYOR-Suite with downloadable example files and example scripts. Additional requirements on file inputs are available through the tutorial and are also described below:

**Installation**

The PATH-SURVEYOR Suite can be downloaded (cloned) and installed through the GitHub repository. The downloaded file can be unzipped to a destination folder, which should be set as the working directory or file path. Of note, some of the example files (e.g., gene set files) use relative paths, so the program may fail to identify the file if a working directory is not properly set.

To get started, a package installation script is provided, which can be run to set up the R environment for the analysis. The Suite was developed in R version 4.1, and while most of the packages and utilities can run on older versions, R v4.1 is required for immune deconvolution. The immunedeconv R package is optional. Requirements associated with its setup can be reviewed here <https://github.com/omnideconv/immunedeconv#standard-r-package>.

- Install PATH-SURVEYOR Suite GitHub repository
  - git clone [https://github.com/shawlab-moffitt/PATH-SURVEYOR-Suite.git](https://github.com/shawlab-moffitt/DRPPM-PATH-SURVEIOR-Suite.git)
  - Download and unzip repository [https://github.com/shawlab-moffitt/PATH-SURVEYOR-Suite/archive/refs/heads/main.zip](https://github.com/shawlab-moffitt/DRPPM-PATH-SURVEIOR-Suite/archive/refs/heads/main.zip)
- Set working directory to PATH-SURVEYOR-Suite folder
- Install required R packages
  - Suite of tools was built on R version 4.1
  - R script for package installation is provided in the “1-Getting_Started” folder

**Key Input Files**

The following tab-delimited input files are necessary to run the Shiny app:

1. **Gene expression file** with gene expression profiling data of patient samples. The gene symbols are in the first column and the sample names are in the first-row header.
2. **Clinical meta information file** that consists of a matrix with the first column the sample name. Following columns contains clinical information, such as event and time-to-event information are required. Other clinical covariates or patient scores (such as immune infiltrate scores) can be included.
3. **Clinical feature parameter file** consists of a two-column file with the first column matching feature names from the “Clinical meta information file.” The second column containing the column type that defines (mandatory input are noted below):
   1. **SampleName** (mandatory)**:** Contains sample names matching the expression data
   2. **SurvivalTime** (mandatory)**:** Contains the overall survival time in days for the samples (can be other types of survival)
   3. **SurvivalID** (mandatory)**:** Contains the survival ID for the samples, should be in a 0/1 format, 0 for alive/no event or 1 for dead/event (can be other types of survival)
   4. **SampleGroup** (optional)**:** Higher level grouping of patient samples
   5. **Feature** (optional)**:** Clinical or non-clinical features that can be included in the Cox-hazard analysis model.

**Required Files**

Here, we provide examples of formatting requirements of the input files. Users must include an expression matrix, clinical meta information, and a clinical feature parameter file. Examples of all required files are provided in the GitHub repository using PAN ICI iAtlas study data, and their formats can be replicated using the guide below.

- Gene Expression File
  - Tab-delimited matrix with gene symbols in the first column and sample names as the first-row header
  - Remove duplicate gene symbols
  - Depending on the size, users may want to remove lowly expressed genes to reduce load time


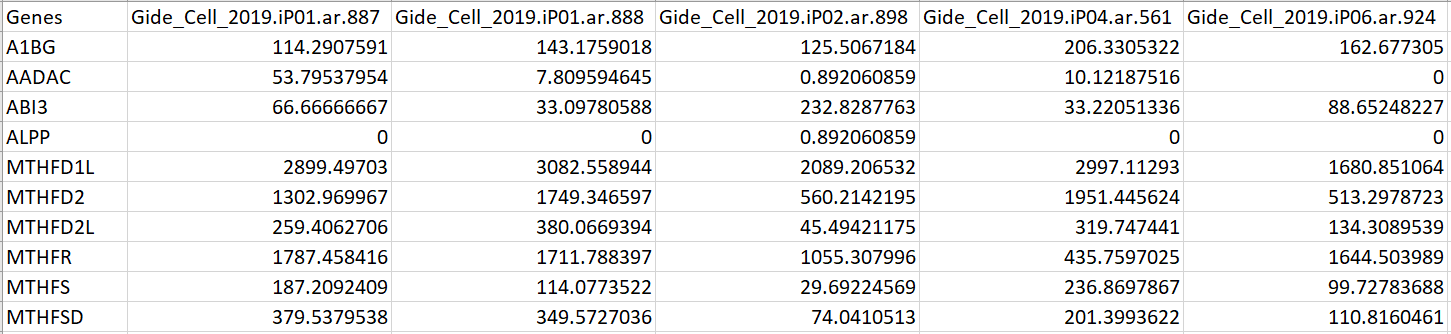


- Clinical Meta Information File
  - Tab-delimited file with the first column requires names matching the samples from the expression matrix. Key columns include **events** (e.g., OS, RFS) and **time-to-event** (e.g., days, months, years). Additional covariates can be included as covariates or pre-processed scores.


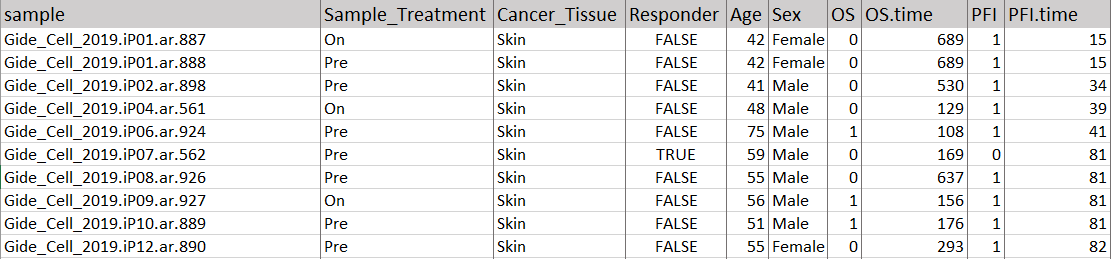


- Clinical Parameter Feature File
  - Tab-delimited two-column file where the first column consists of column names of the “Clinical Meta Information File” and the second column defines the column type
    - **SampleName** (mandatory)**:** Contains sample names matching the expression data
    - **SurvivalTime** (mandatory)**:** Contains the overall survival time in days for the samples (can be other types of survival)
    - **SurvivalID** (mandatory)**:** Contains the survival ID for the samples, should be in a 0/1 format, 0 for alive/no event or 1 for dead/event (can be other types of survival)
    - **SampleGroup** (optional)**:** Higher level grouping of patient samples
    - **Feature** (optional)**:** Clinical or non-clinical features that can be included in the Cox-hazard analysis model.


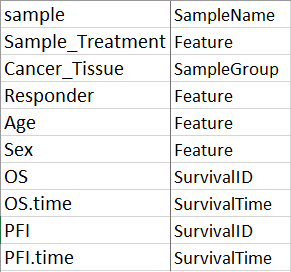


**PATH-SURVEYOR: Interactive Mode**

The Interactive R Shiny application can be set up through the **app.R** script found in the **2-PATH-SURVEYOR-InteractiveApp** folder. This application takes the input of a gene expression matrix, clinical metainformation, and clinical parameter feature as described in the required files section. As an example, the app.R script references several input files necessary to perform pathway-level survival analysis in a patient treated with check-point inhibition from iAtlas. There is a section with advanced user input options, which is further described further in GitHub. File paths to gene sets and R markdown are also provided. The app can be executed with the runApp() function or by clicking the “Run App” button in the R Studio interface.

<https://github.com/shawlab-moffitt/PATH-SURVEYOR-Suite/tree/main/2-PATH-SURVEYOR-Interactive-App>

- Script
  - PATH-SURVEYOR-Suite/2-PATH-SURVEYOR-InteractiveApp/app.R
- Input
  - Project Name: A descriptive name for your project/data
  - File inputs: Supply the path and file name of the expression matrix, clinical meta information, and clinical meta feature parameter files
  - Advanced User Input
    - Pre-set UI input options to be chosen upon startup, further described in GitHub README
  - Gene Set Database and Markdown files
    - Available through the GitHub repository.

**PATH-SURVEYOR: Pipeline Mode**

The PATH-SURVEYOR Pipeline can be run on the command line or in R Studio. The setup requires a two-column, tab-delimited parameter file, which contains the parameter names in the first column and the file paths and options to guide the script in the second column. A guide to generating this file can be found within the GitHub repository ([https://github.com/shawlab-moffitt/PATH-SURVEYOR-Pipeline#parameter-file](https://github.com/shawlab-moffitt/DRPPM-PATH-SURVEIOR-Pipeline#parameter-file)). This pipeline can be run at the single gene or pathway level, identifying genes or pathways associated with hazard ratio. When running the script in R studio, the parameter file path can be included at the beginning of the Coxh_Ranking.R script and executed locally or through the “Rscript” command with the parameter file path as input. Depending on the sample size or gene set size, this script can take between 5 minutes and 24 hours to run. Significant pathways identified by this pipeline can be used as input for the Jaccard Connectivity App. The output list of Genes ranked based on hazard ratio can be used as input to the Hazard Ratio Ranked GSEA app.

<https://github.com/shawlab-moffitt/PATH-SURVEYOR-Suite/tree/main/3-PATH-SURVEYOR-Pipeline>

- Script
  - Scripts to run in R Studio and in a command line interface are found here: PATH-SURVEYOR-Suite/3-PATH-SURVEYOR-Pipeline/
- Input
  - Parameter File: Tab-delimited two-column file containing input file paths and run parameters described in the GitHub [https://github.com/shawlab-moffitt/PATH-SURVEYOR-Pipeline#parameter-file](https://github.com/shawlab-moffitt/DRPPM-PATH-SURVEIOR-Pipeline#parameter-file)
    - Pathway Level: When ranking gene set pathways according to Cox proportional hazards, a gene set file and name are required. Users can include a number of top pathways ranked on significance, and a Jaccard connectivity matrix will be included in the output.
    - Gene Level: When ranking individual genes, no gene set file is required, though, if one is included, users can select to perform GSEA with a hazard ratio ranked list of genes upon Cox regression completion.


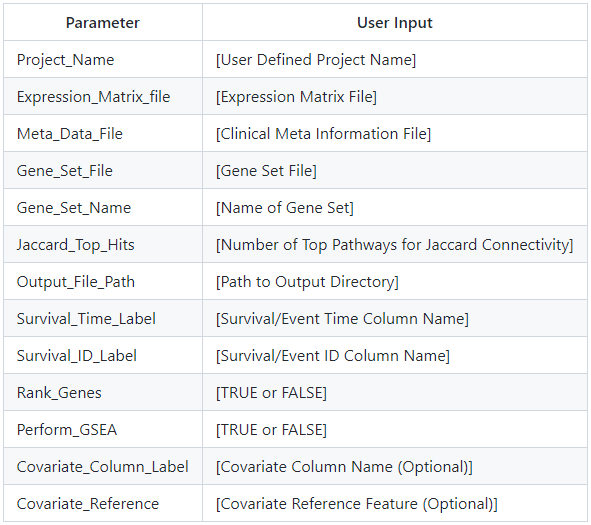


- Output
  - ssGSEA score table (gene set file required)
  - Median Cut-Point table
  - Cox Proportional Hazard Regression output for all pathways or genes
  - Jaccard Connectivity Matrix (gene set file required)
  - Hazard Ratio Ranked GSEA (gene set file required)

**PATH-SURVEYOR: Jaccard Connectivity**

The Jaccard Connectivity R Shiny application can be initiated through the app.R script in the 4-DRPPM-Jaccard_Connectivity_App folder. The top of the app.R script has a section that requires the user to fill out the path to the comprehensive gene set file, which is available through the GitHub page. This file is used in the back end of the application and contains the genes that are found within the gene sets. Once the app is deployed, the input file for analysis is the output file from PATH-SURVEYOR Pipeline pathway analysis.

<https://github.com/shawlab-moffitt/PATH-SURVEYOR-Suite/tree/main/4-Pathway-Connectivity-App>

- Script
  - PATH-SURVEYOR-Suite/4-Pathway-Connectivity-App/app.R
- Input
  - Gene Set File:
    - GeneSet_Data/Comprehensive_GeneSet.Rdata
  - User-Derived (Input after app startup)
    - CoxPH output file from PATH-SURVEYOR Pipeline pathway analysis
    - CoxPH output file from PATH-SURVEYOR Pipeline gene analysis
      - For gene cluster annotation (Optional)
    - GMT file of gene set pathways

**PATH-SURVEYOR: Hazard Ratio Ranked GSEA**

Hazard Ratio Ranked GSEA R Shiny Application can be initiated through the app.R script in the 5-DRPPM-Hazard_Ratio_Ranked_GSEA_App folder. The top of the app.R script has a line that takes the path to the comprehensive gene set file, which is provided in GitHub. The primary input is a Cox proportional hazards rank of genes derived from the PATH-SURVEYOR Pipeline. This file contains a variety of gene sets to perform GSEA, including MSigDB, LINCS L1000, Cell Marker. A murine version of MSigDB and Cell Marker is also available. Upload of a user-custom gene set is also available.

<https://github.com/shawlab-moffitt/PATH-SURVEYOR-Suite/tree/main/5-PreRanked-HazardRatio-GSEA-App>

- Script
  - PATH-SURVEYOR-Suite/5-PreRanked-HazardRatio-GSEA-App/app.R
- Input
  - Gene Set File:
    - GeneSet_Data/GeneSets.zip
  - User-Derived (Input upon app startup)
    - CoxPH output file from PATH-SURVEYOR Pipeline gene analysis

**Immune Deconvolution Pre-Processing**

While Immune Deconvolution can be inferred in real-time, we also provide the option to pre-process this information for speed-up purposes. To perform the immune deconvolution pre-processing, the top section of the script is annotated as “User-Input”, which requires user editing to include proper file names and paths. Other parameters include “project name” is used for naming the output files and the “output path” directs the path of the output files. The annotated immune deconvolution methods can be turned on or off by the user (by specifying TRUE or FALSE). After specifying the inputs above, the script can be executed as a local job in R studios. Outputs include an immune deconvolution score matrix, a clinical meta parameter file, and an updated clinical meta file with appended immune deconvolution scores. These output files can be used as input to the PATH-SURVEYOR App for the point-and-click analysis. Further information on this script is available in the README.

- Script
  - 1-Getting_Started/2-Immune_Deconvolution/Immune_Deconvolution.R
  - Only available for R version 4.1 or greater
- Input
  - ProjectName: A descriptive name for your project/data
  - File inputs: Supply the path and file name or your expression matrix, clinical meta information, and clinical meta feature parameter files
  - Output_Path: Provide a path to write the output files to
  - Immune Deconvolution Methods: Indicate with TRUE or FALSE which methods to run
- Output
  - Updated clinical meta information and clinical meta feature parameter file, which can be used as input to the interactive Shiny app

**References**

1. Borcherding N, Bormann NL, Voigt AP, Zhang W: **TRGAted: A web tool for survival analysis using protein data in the Cancer Genome Atlas**. *F1000Res* 2018, **7**:1235.

2. Chandrashekar DS, Bashel B, Balasubramanya SAH, Creighton CJ, Ponce-Rodriguez I, Chakravarthi B, Varambally S: **UALCAN: A Portal for Facilitating Tumor Subgroup Gene Expression and Survival Analyses**. *Neoplasia* 2017, **19**(8):649-658.

3. Dereli O, Oguz C, Gonen M: **Path2Surv: Pathway/gene set-based survival analysis using multiple kernel learning**. *Bioinformatics* 2019, **35**(24):5137-5145.

4. Jeuken GS, Tobin NP, Kall L: **Survival analysis of pathway activity as a prognostic determinant in breast cancer**. *PLoS Comput Biol* 2022, **18**(3):e1010020.

5. Rupji M, Zhang X, Kowalski J: **CASAS: Cancer Survival Analysis Suite, a web based application**. *F1000Res* 2017, **6**:919.

6. Pak K, Oh SO, Goh TS, Heo HJ, Han ME, Jeong DC, Lee CS, Sun H, Kang J, Choi S *et al*: **A User-Friendly, Web-Based Integrative Tool (ESurv) for Survival Analysis: Development and Validation Study**. *J Med Internet Res* 2020, **22**(5):e16084.

7. Lanczky A, Gyorffy B: **Web-Based Survival Analysis Tool Tailored for Medical Research (KMplot): Development and Implementation**. *J Med Internet Res* 2021, **23**(7):e27633.

8. Dwivedi B, Mumme H, Satpathy S, Bhasin SS, Bhasin M: **Survival Genie, a web platform for survival analysis across pediatric and adult cancers**. *Sci Rep* 2022, **12**(1):3069.
