# Supplementary figures and images for: PATH-SURVEYOR: pathway level survival enquiry for immuno-oncology and drug repurposing
Source: BMC Bioinformatics. 2023 Jun 28;24:266. doi: 10.1186/s12859-023-05393-y (PMC10303868; doi:10.1186/s12859-023-05393-y)

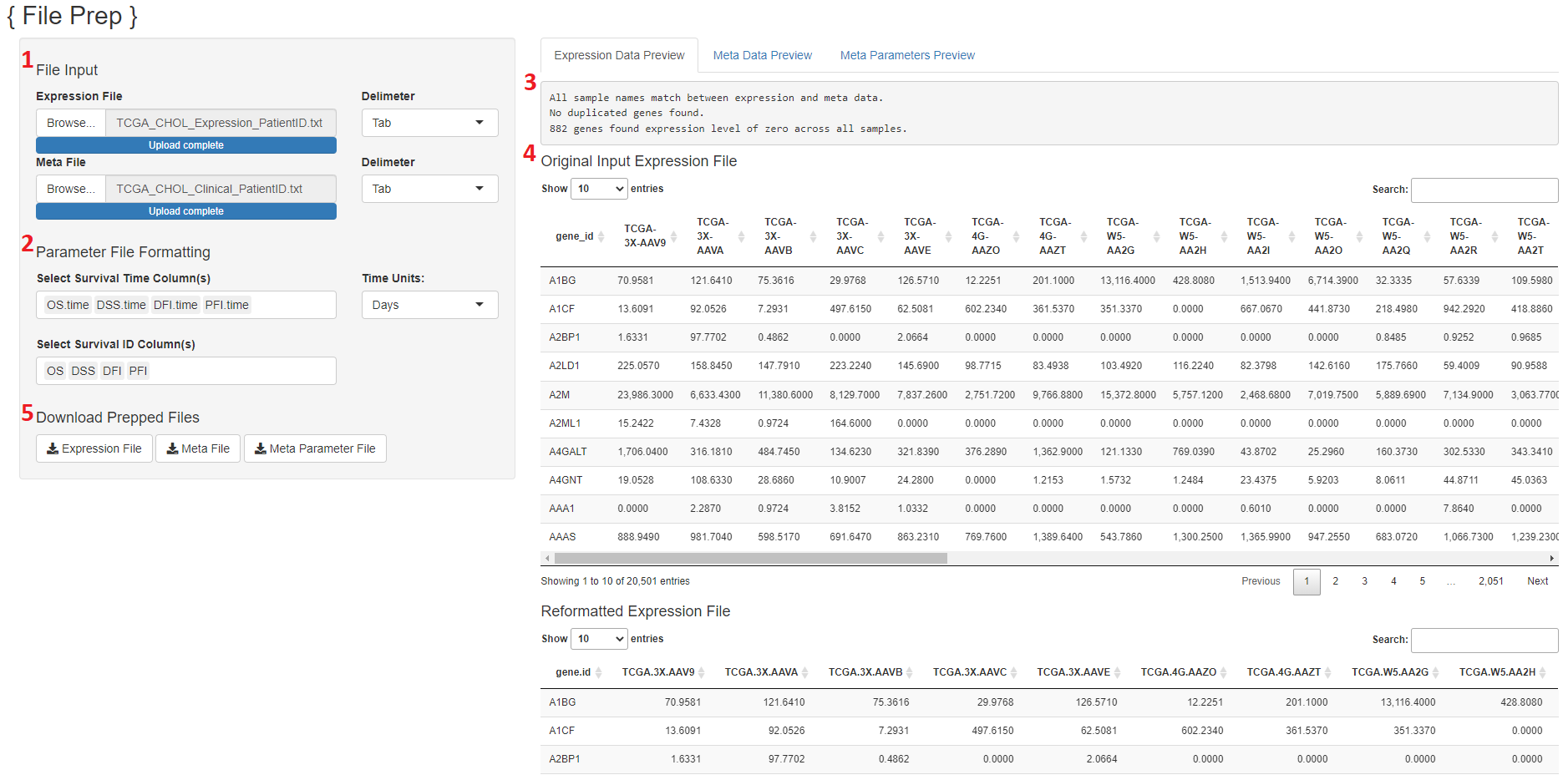

Supplement: Supplementary file 10 — Additional file 10. PATH-SURVEYOR-Suite-main.zip. [file 12859_2023_5393_MOESM10_ESM.zip › PATH-SURVEYOR-Suite-main/1-Getting_Started/2-FilePrep/Example_UI_Screenshots/PATH_SURVEYOR_FilePrepApp1.PNG]

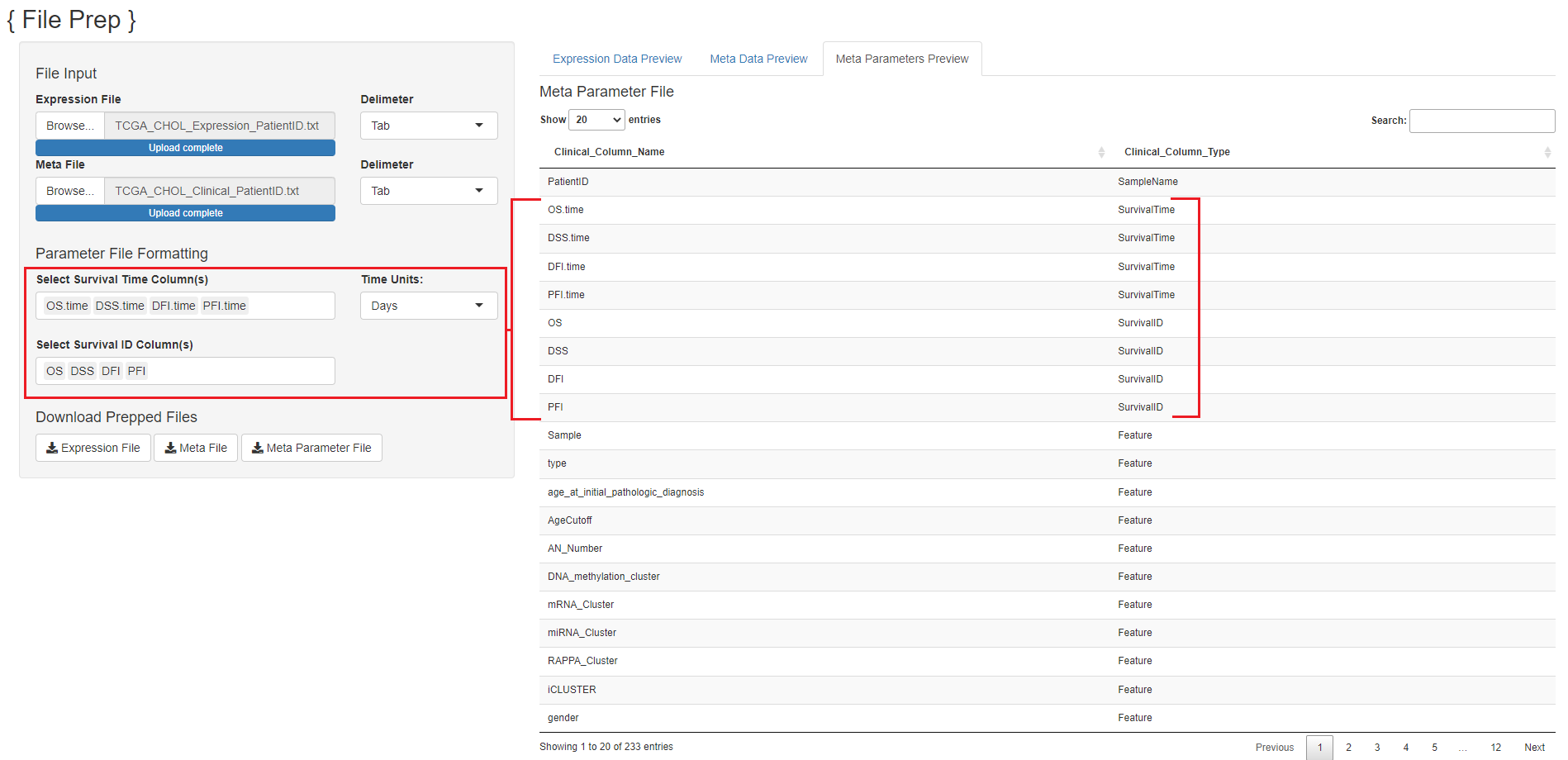

Supplement: Supplementary file 10 — Additional file 10. PATH-SURVEYOR-Suite-main.zip. [file 12859_2023_5393_MOESM10_ESM.zip › PATH-SURVEYOR-Suite-main/1-Getting_Started/2-FilePrep/Example_UI_Screenshots/PATH_SURVEYOR_FilePrepApp2.PNG]

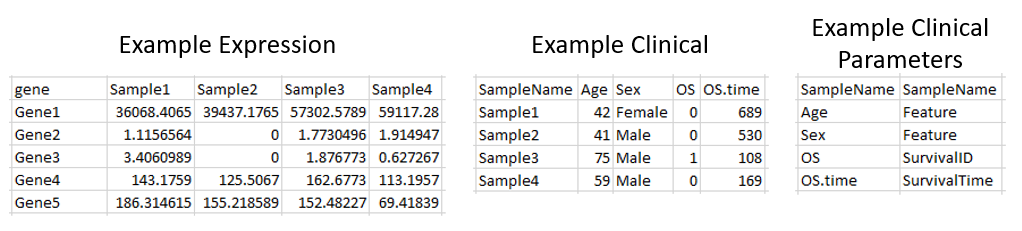

Supplement: Supplementary file 10 — Additional file 10. PATH-SURVEYOR-Suite-main.zip. [file 12859_2023_5393_MOESM10_ESM.zip › PATH-SURVEYOR-Suite-main/2-PATH-SURVEYOR-Interactive-App/App_Demo_Pictures/ExampleData.png]

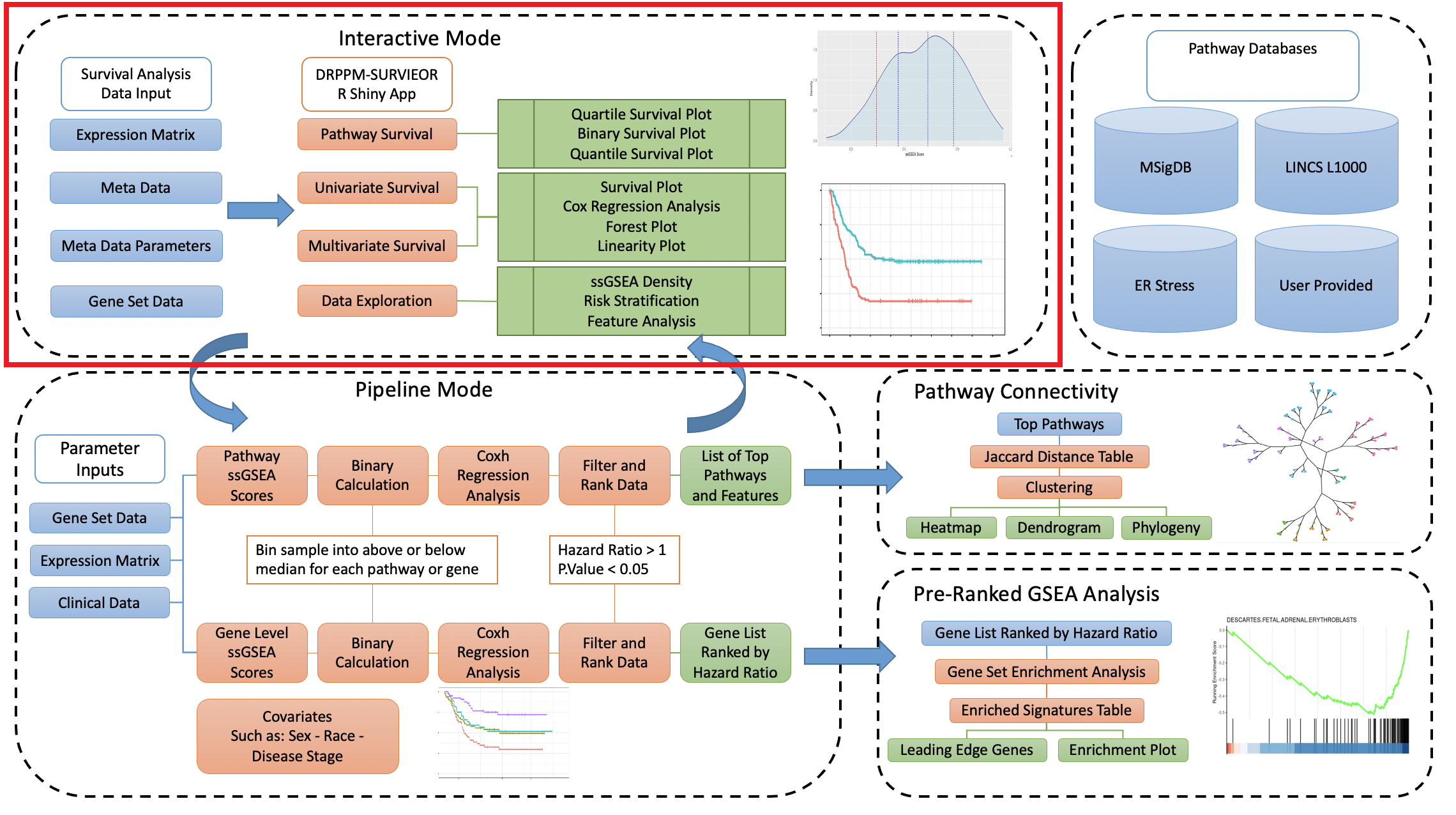

Supplement: Supplementary file 10 — Additional file 10. PATH-SURVEYOR-Suite-main.zip. [file 12859_2023_5393_MOESM10_ESM.zip › PATH-SURVEYOR-Suite-main/2-PATH-SURVEYOR-Interactive-App/App_Demo_Pictures/FlowChart_InteractiveMode.png]

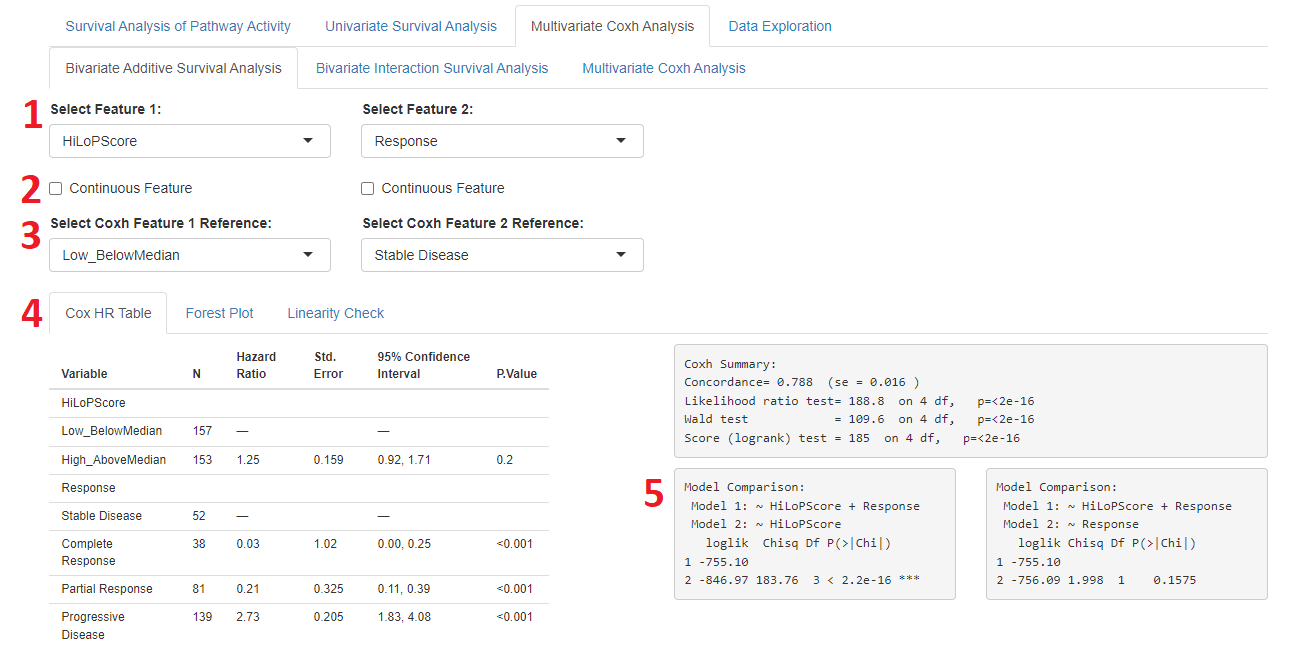

Supplement: Supplementary file 10 — Additional file 10. PATH-SURVEYOR-Suite-main.zip. [file 12859_2023_5393_MOESM10_ESM.zip › PATH-SURVEYOR-Suite-main/2-PATH-SURVEYOR-Interactive-App/App_Demo_Pictures/MainPanel_BivarAdd_Survival.png]

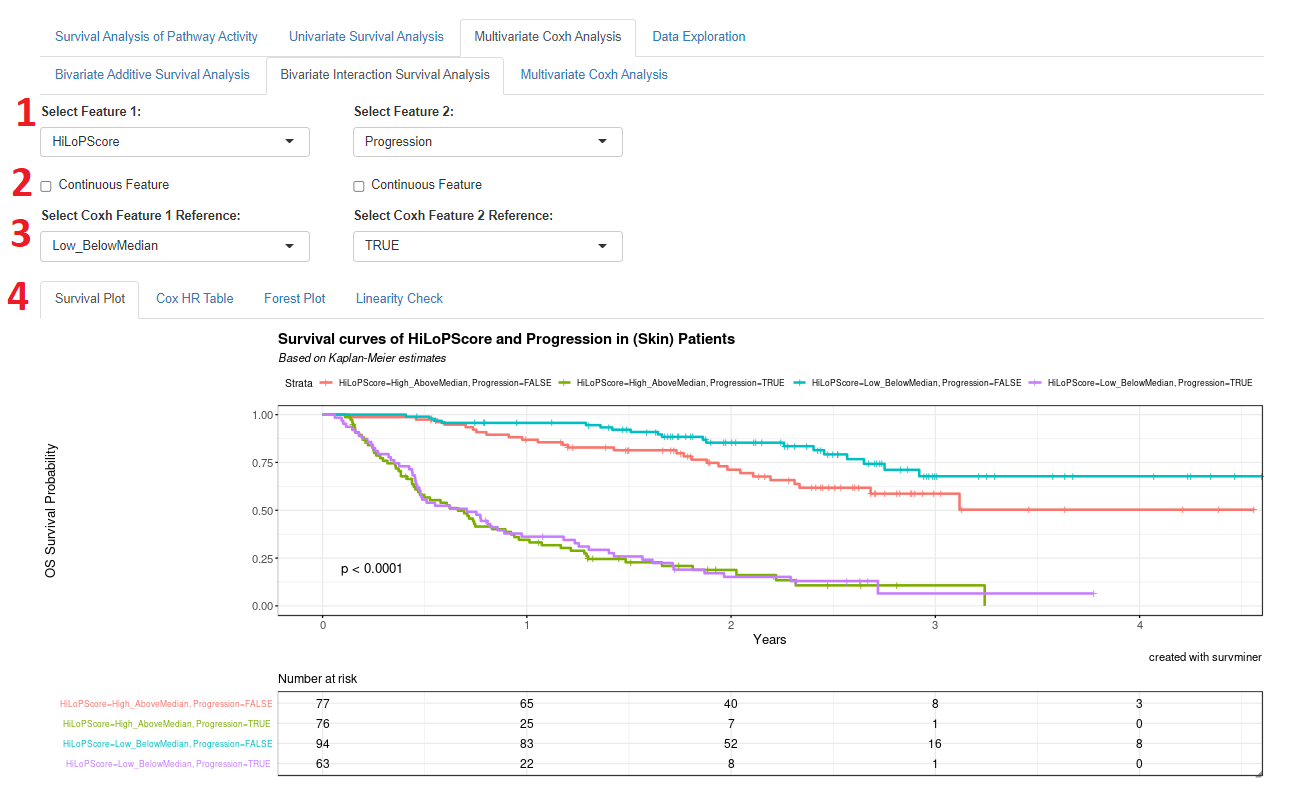

Supplement: Supplementary file 10 — Additional file 10. PATH-SURVEYOR-Suite-main.zip. [file 12859_2023_5393_MOESM10_ESM.zip › PATH-SURVEYOR-Suite-main/2-PATH-SURVEYOR-Interactive-App/App_Demo_Pictures/MainPanel_Bivar_Inter_Survival.png]

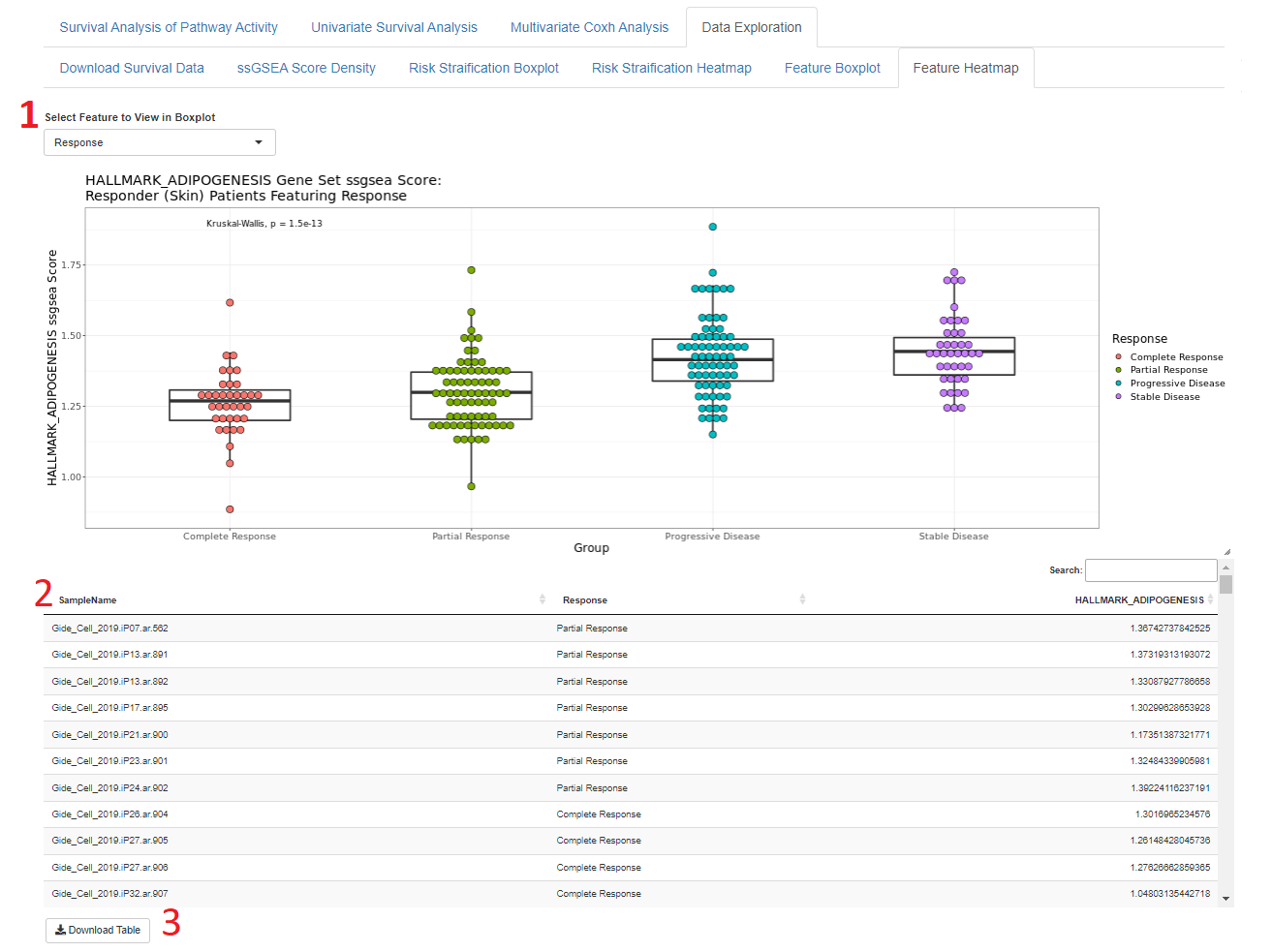

Supplement: Supplementary file 10 — Additional file 10. PATH-SURVEYOR-Suite-main.zip. [file 12859_2023_5393_MOESM10_ESM.zip › PATH-SURVEYOR-Suite-main/2-PATH-SURVEYOR-Interactive-App/App_Demo_Pictures/MainPanel_Feature_Boxplot.png]

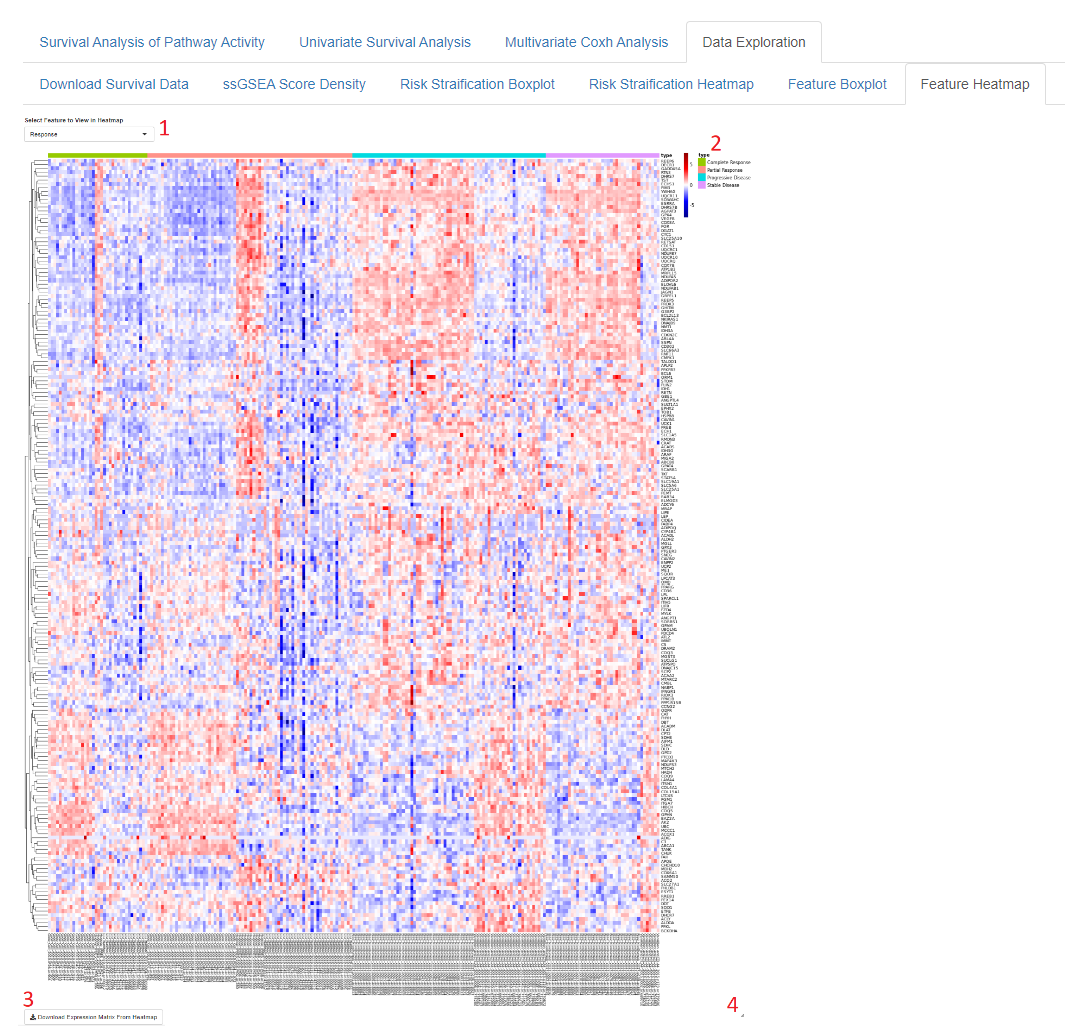

Supplement: Supplementary file 10 — Additional file 10. PATH-SURVEYOR-Suite-main.zip. [file 12859_2023_5393_MOESM10_ESM.zip › PATH-SURVEYOR-Suite-main/2-PATH-SURVEYOR-Interactive-App/App_Demo_Pictures/MainPanel_Feature_Heatmap.png]

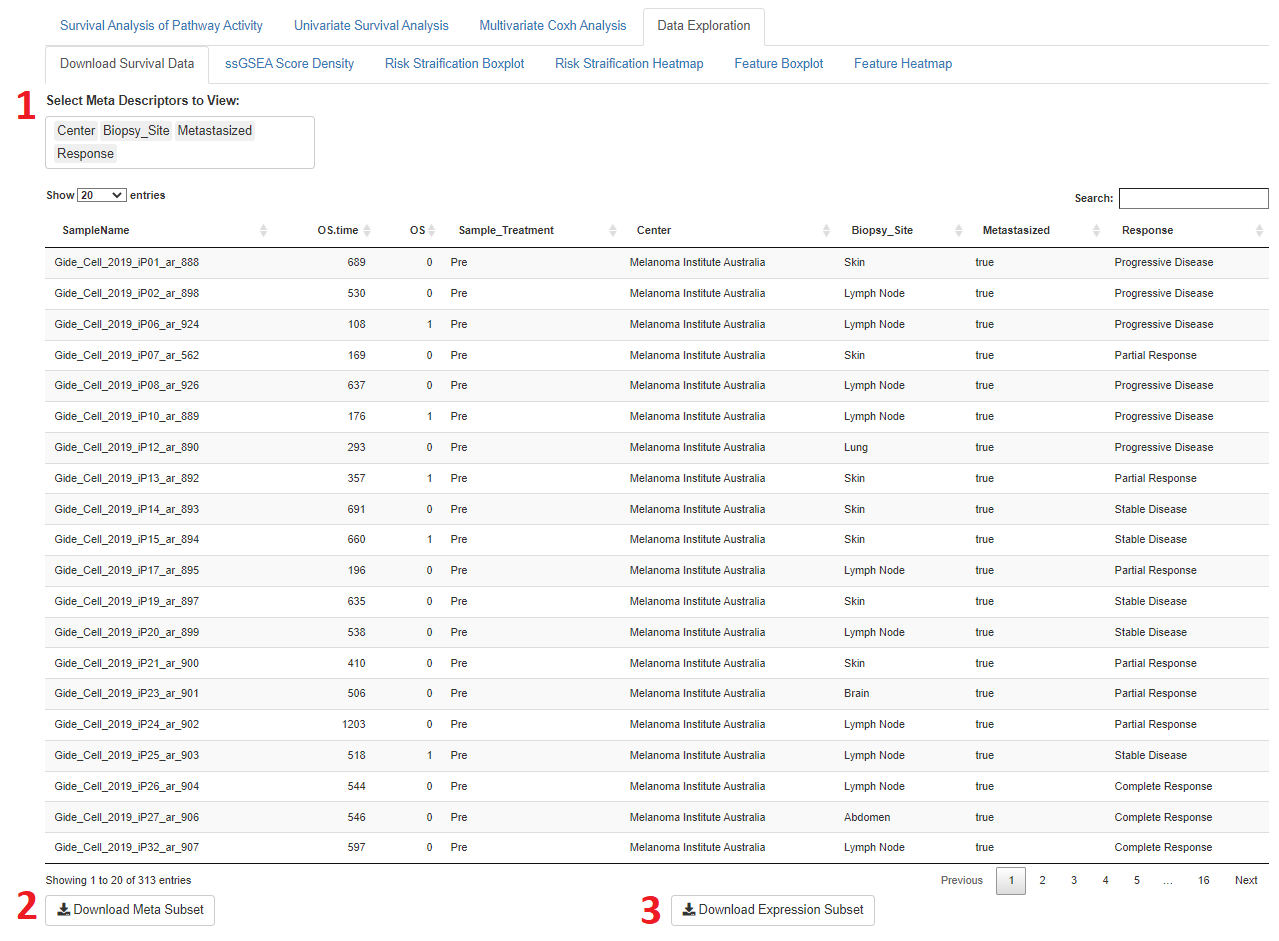

Supplement: Supplementary file 10 — Additional file 10. PATH-SURVEYOR-Suite-main.zip. [file 12859_2023_5393_MOESM10_ESM.zip › PATH-SURVEYOR-Suite-main/2-PATH-SURVEYOR-Interactive-App/App_Demo_Pictures/MainPanel_MetaTable.png]

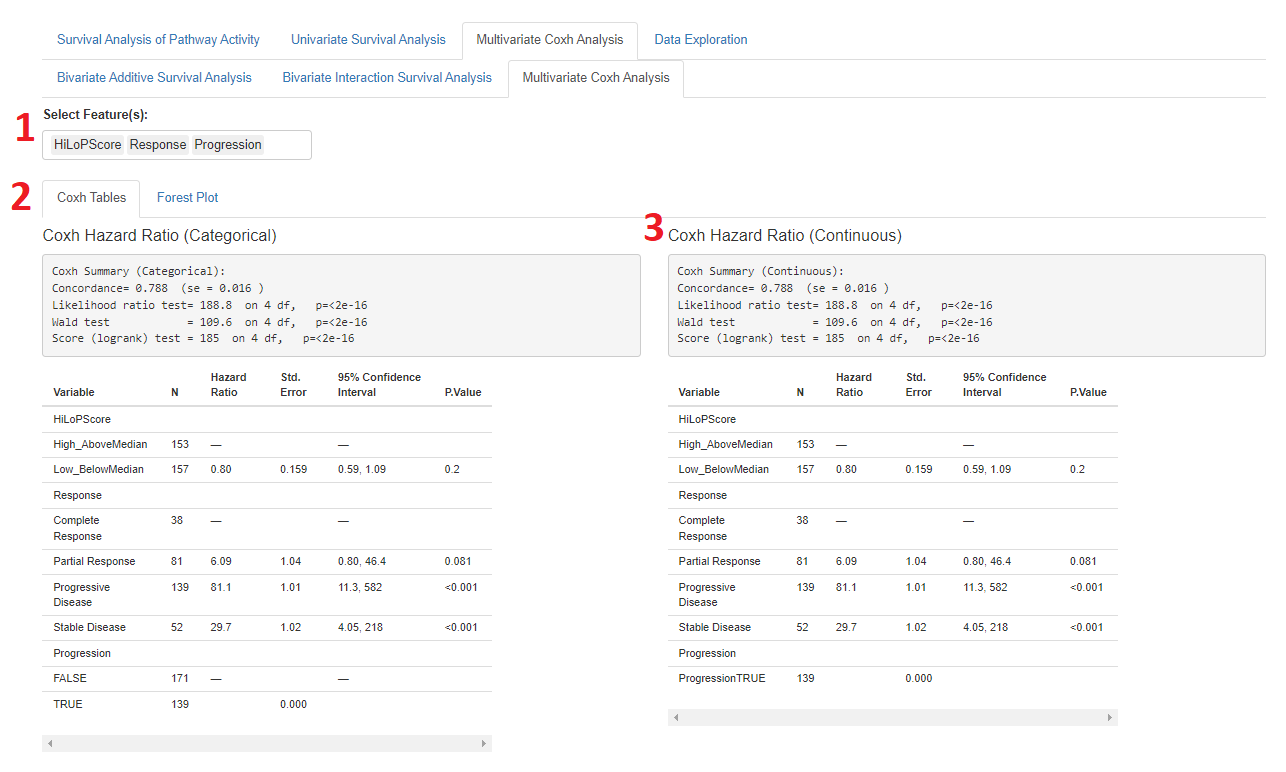

Supplement: Supplementary file 10 — Additional file 10. PATH-SURVEYOR-Suite-main.zip. [file 12859_2023_5393_MOESM10_ESM.zip › PATH-SURVEYOR-Suite-main/2-PATH-SURVEYOR-Interactive-App/App_Demo_Pictures/MainPanel_Multivar_Survival.png]

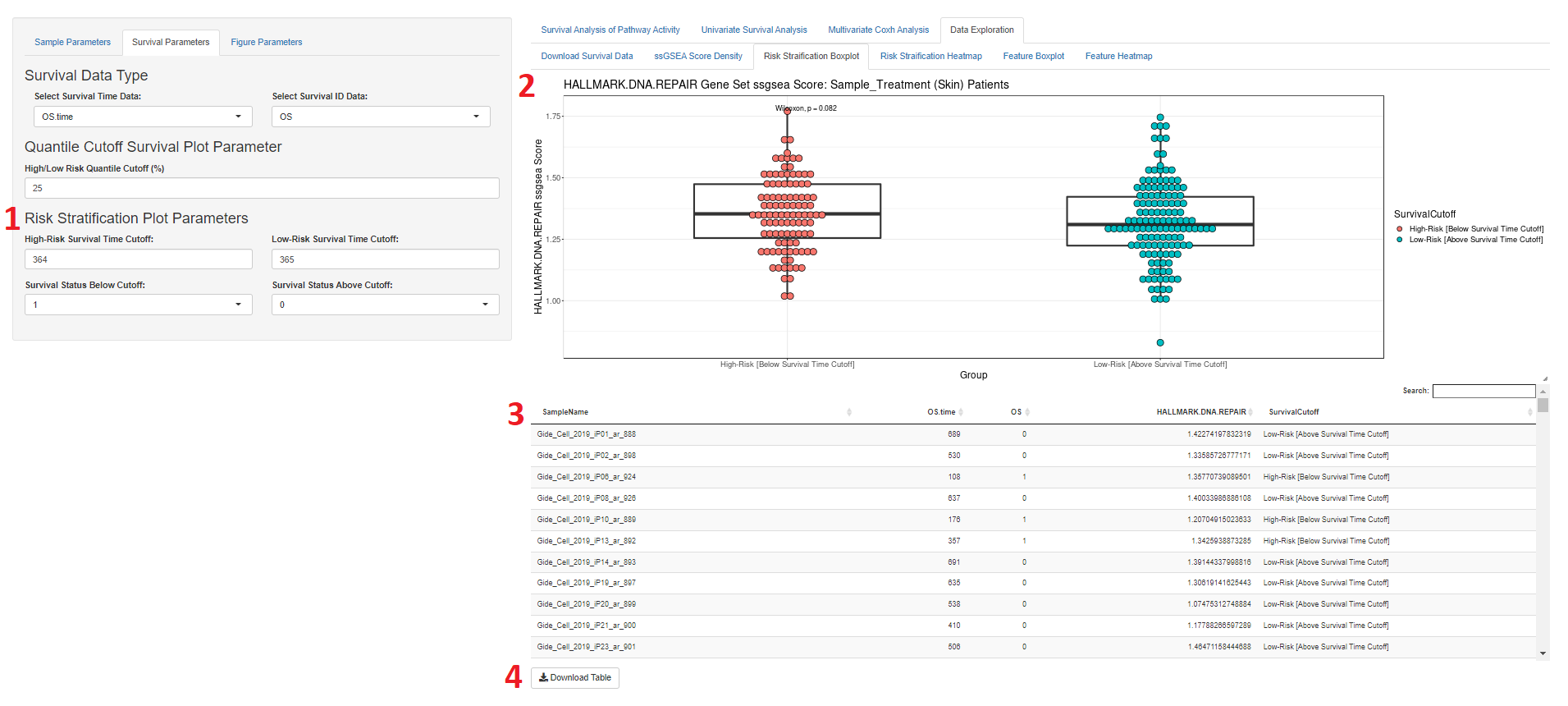

Supplement: Supplementary file 10 — Additional file 10. PATH-SURVEYOR-Suite-main.zip. [file 12859_2023_5393_MOESM10_ESM.zip › PATH-SURVEYOR-Suite-main/2-PATH-SURVEYOR-Interactive-App/App_Demo_Pictures/MainPanel_RiskStrat_BoxPlot.png]

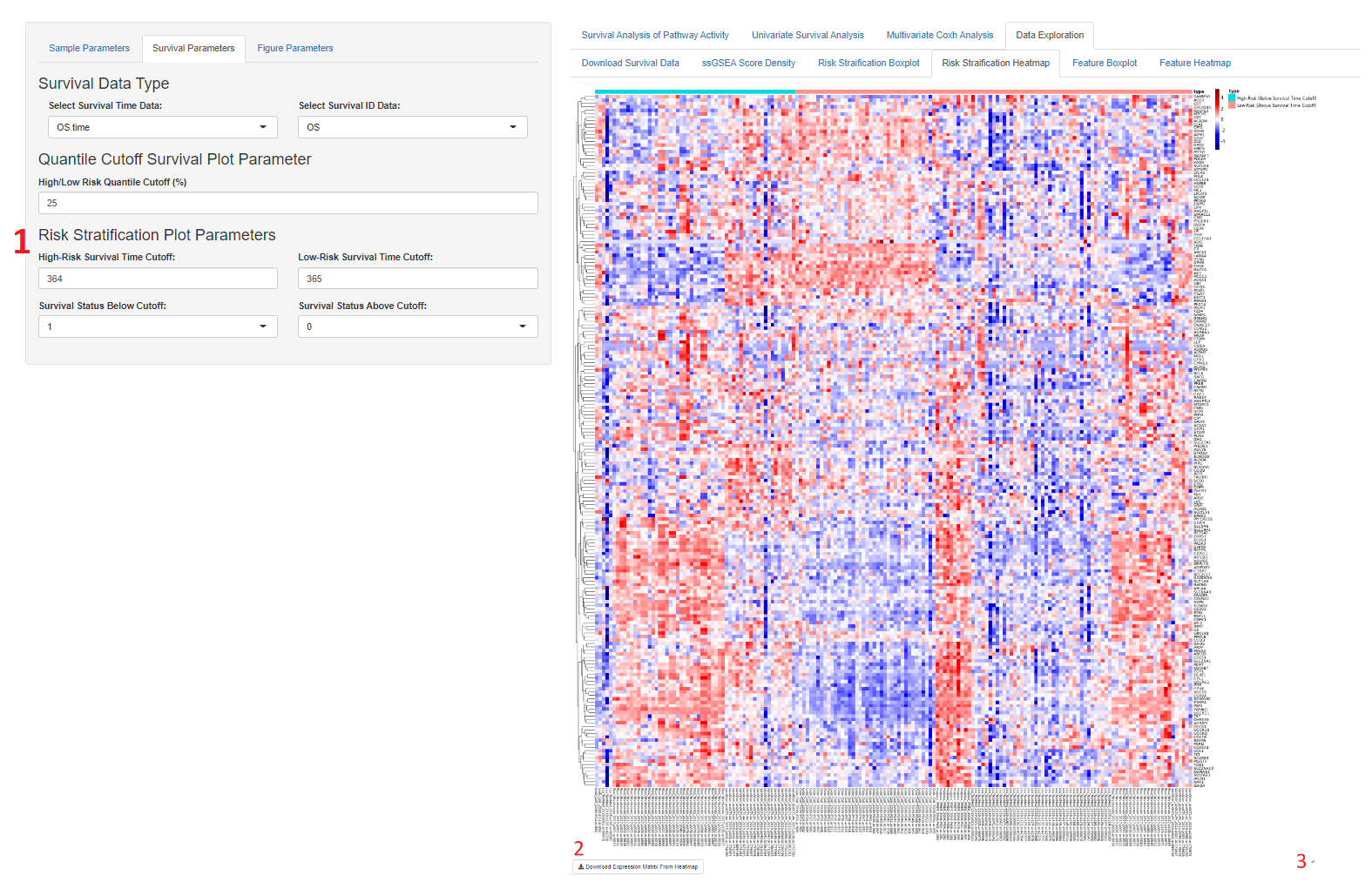

Supplement: Supplementary file 10 — Additional file 10. PATH-SURVEYOR-Suite-main.zip. [file 12859_2023_5393_MOESM10_ESM.zip › PATH-SURVEYOR-Suite-main/2-PATH-SURVEYOR-Interactive-App/App_Demo_Pictures/MainPanel_RiskStrat_Heatmap.png]

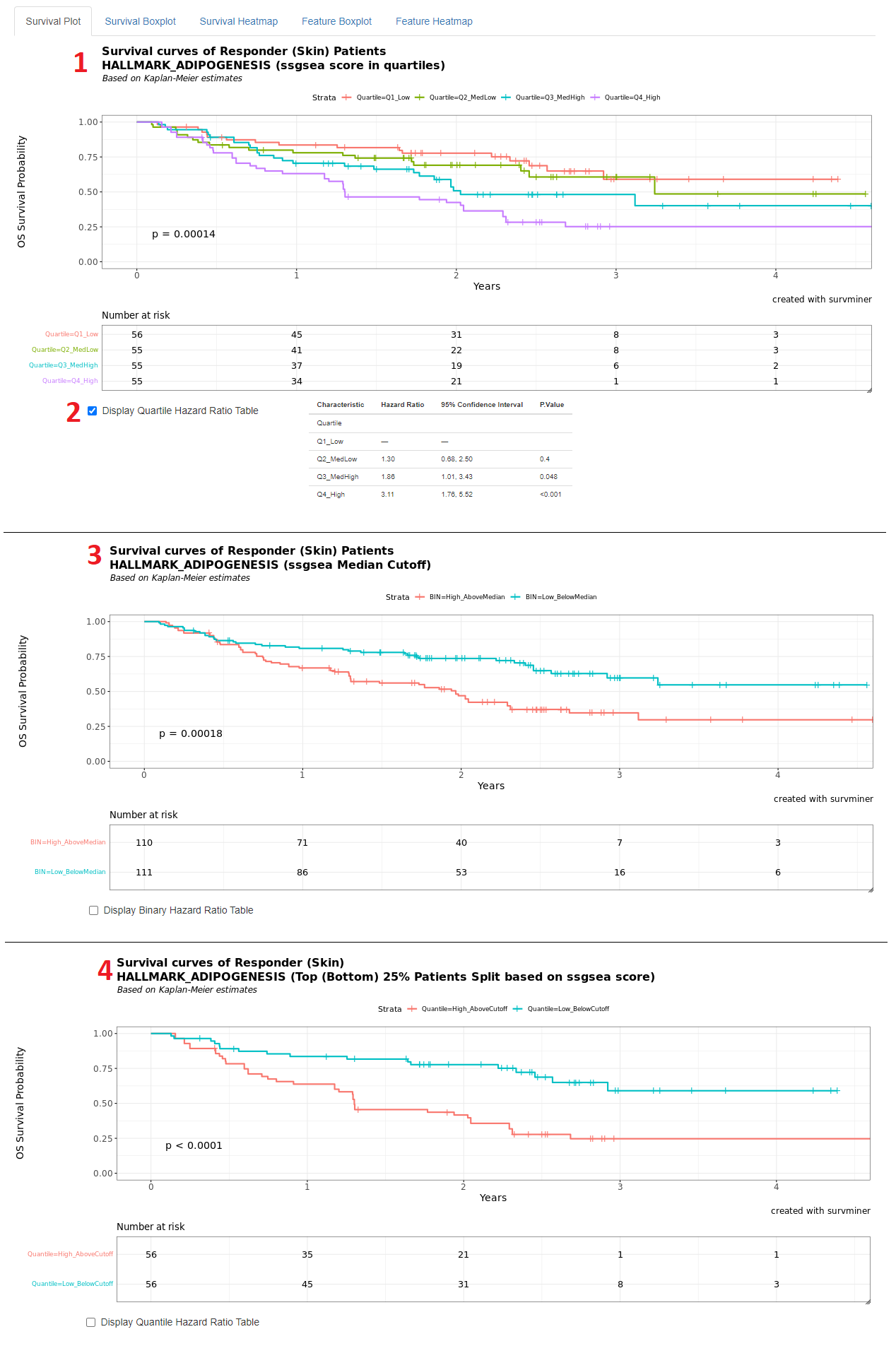

Supplement: Supplementary file 10 — Additional file 10. PATH-SURVEYOR-Suite-main.zip. [file 12859_2023_5393_MOESM10_ESM.zip › PATH-SURVEYOR-Suite-main/2-PATH-SURVEYOR-Interactive-App/App_Demo_Pictures/MainPanel_SurvivalPlot.png]

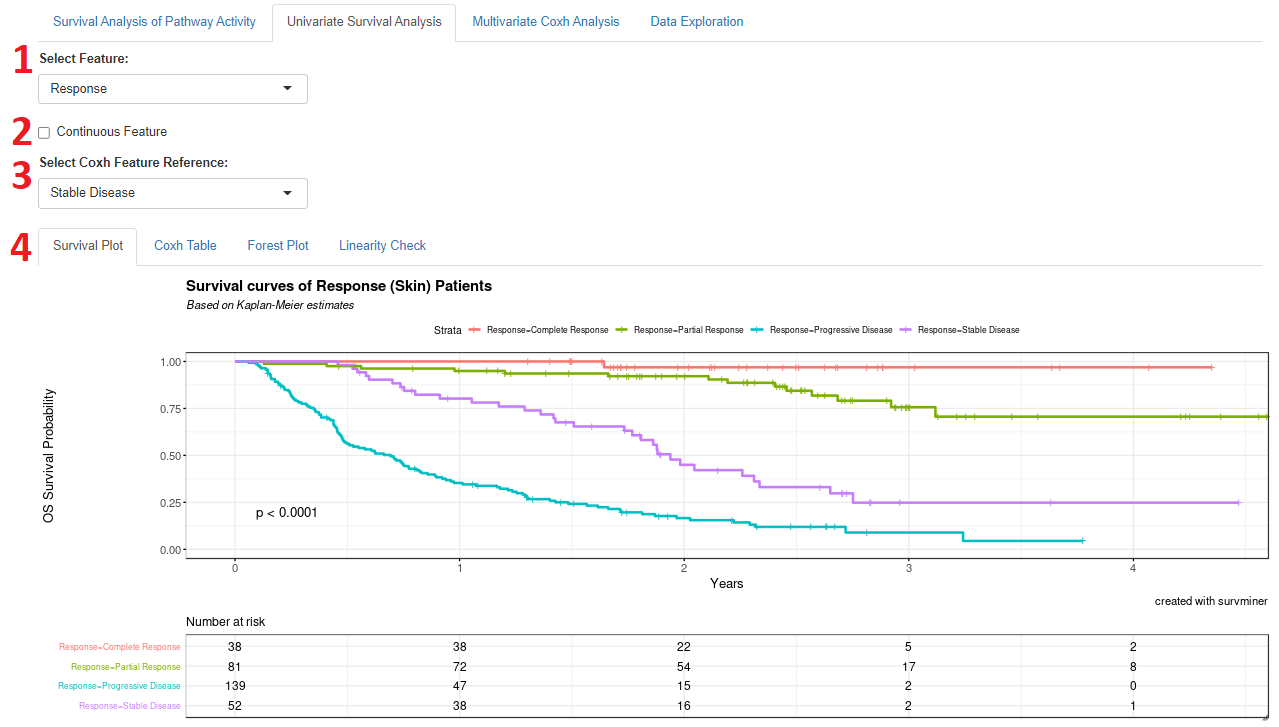

Supplement: Supplementary file 10 — Additional file 10. PATH-SURVEYOR-Suite-main.zip. [file 12859_2023_5393_MOESM10_ESM.zip › PATH-SURVEYOR-Suite-main/2-PATH-SURVEYOR-Interactive-App/App_Demo_Pictures/MainPanel_Univar_Survival.png]

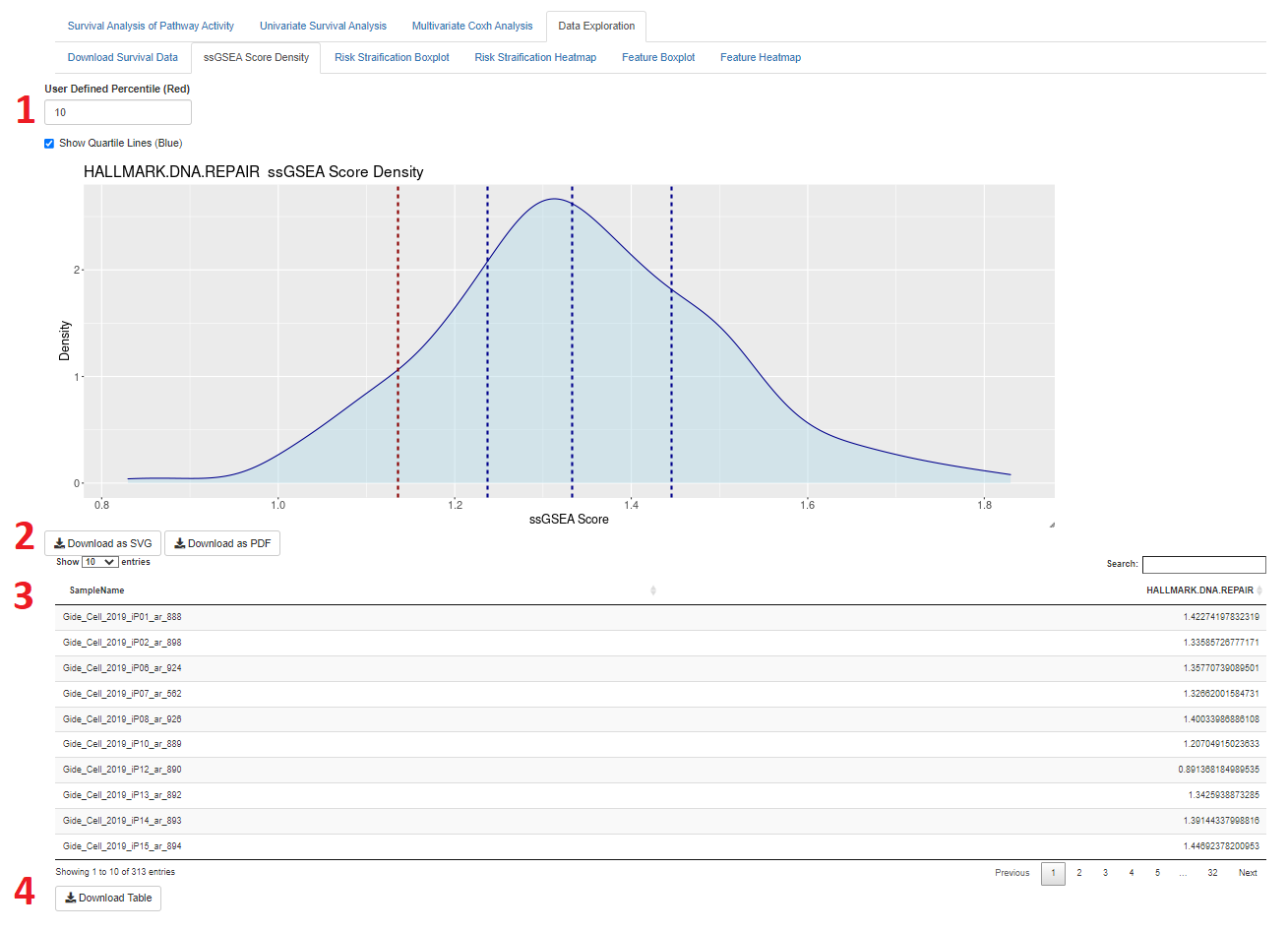

Supplement: Supplementary file 10 — Additional file 10. PATH-SURVEYOR-Suite-main.zip. [file 12859_2023_5393_MOESM10_ESM.zip › PATH-SURVEYOR-Suite-main/2-PATH-SURVEYOR-Interactive-App/App_Demo_Pictures/MainPanel_ssGSEA_Density.png]

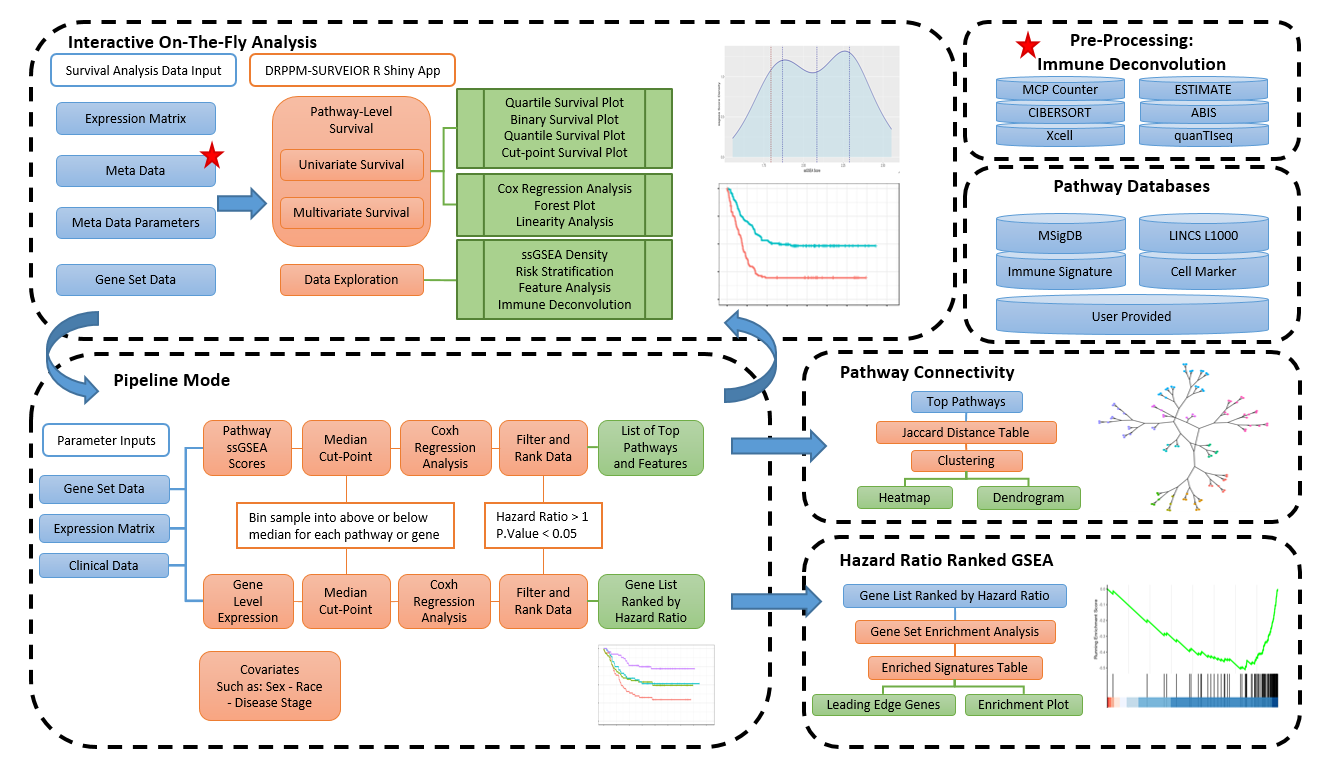

Supplement: Supplementary file 10 — Additional file 10. PATH-SURVEYOR-Suite-main.zip. [file 12859_2023_5393_MOESM10_ESM.zip › PATH-SURVEYOR-Suite-main/2-PATH-SURVEYOR-Interactive-App/App_Demo_Pictures/Main_schematic.PNG]

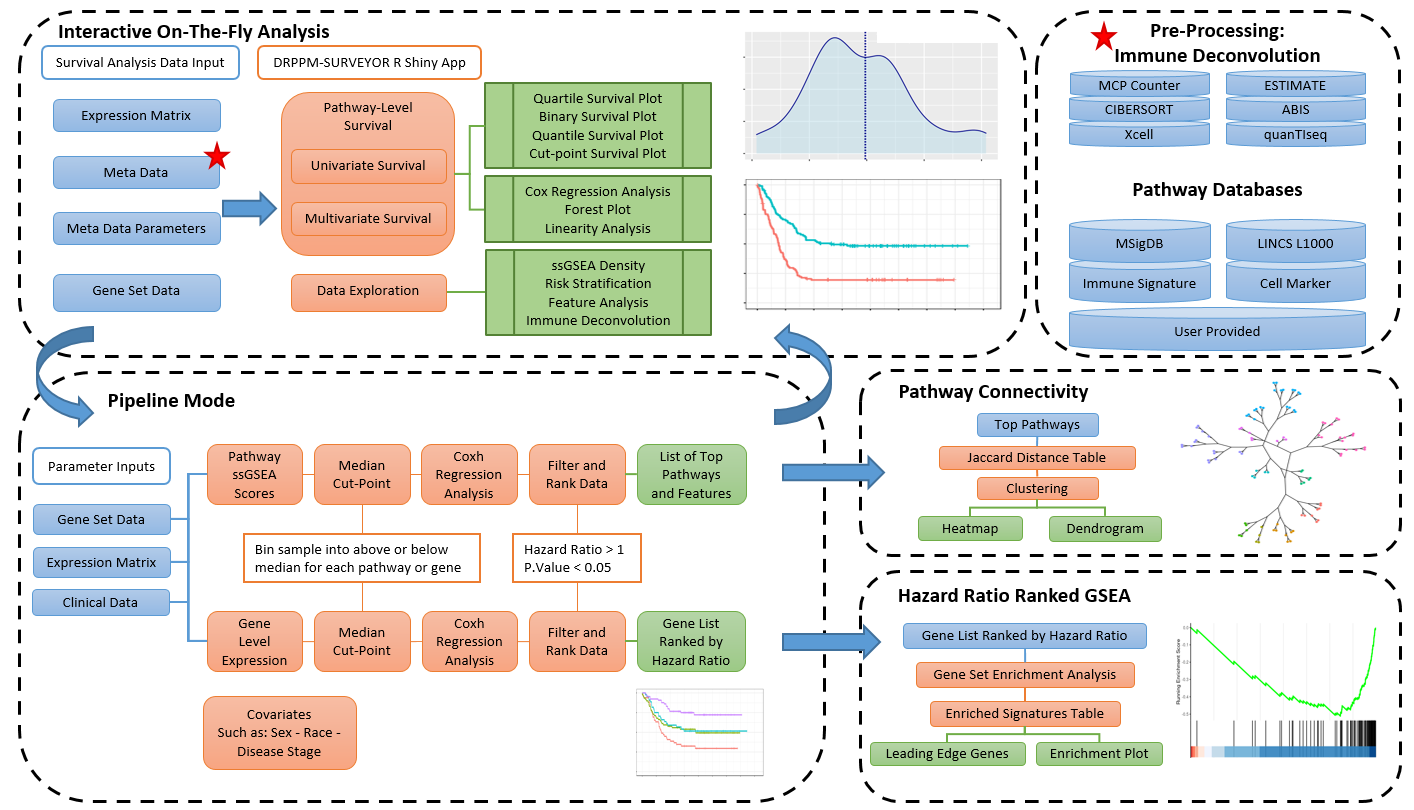

Supplement: Supplementary file 10 — Additional file 10. PATH-SURVEYOR-Suite-main.zip. [file 12859_2023_5393_MOESM10_ESM.zip › PATH-SURVEYOR-Suite-main/2-PATH-SURVEYOR-Interactive-App/App_Demo_Pictures/PATH_SURVEYOR_Main_schematic.PNG]

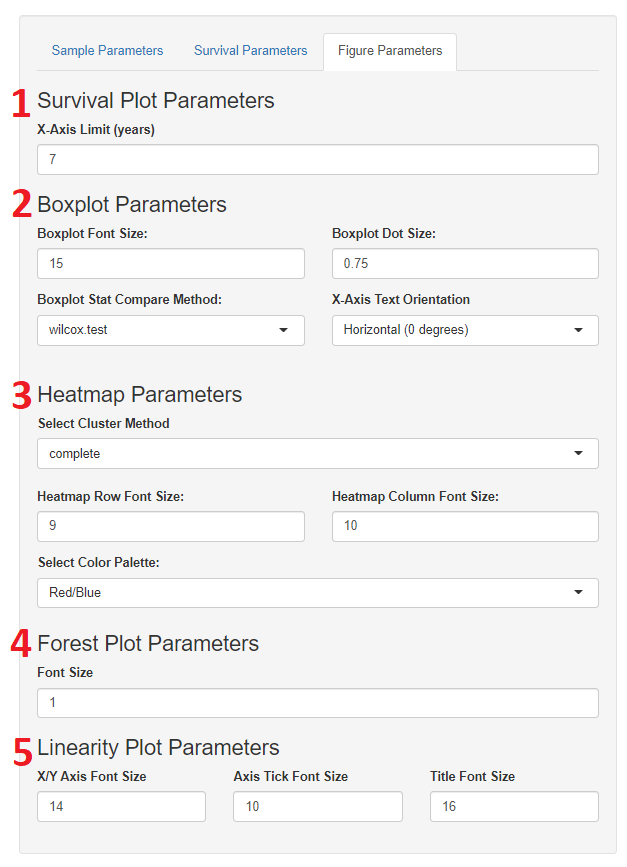

Supplement: Supplementary file 10 — Additional file 10. PATH-SURVEYOR-Suite-main.zip. [file 12859_2023_5393_MOESM10_ESM.zip › PATH-SURVEYOR-Suite-main/2-PATH-SURVEYOR-Interactive-App/App_Demo_Pictures/SideBar_FigureParamaters.png]

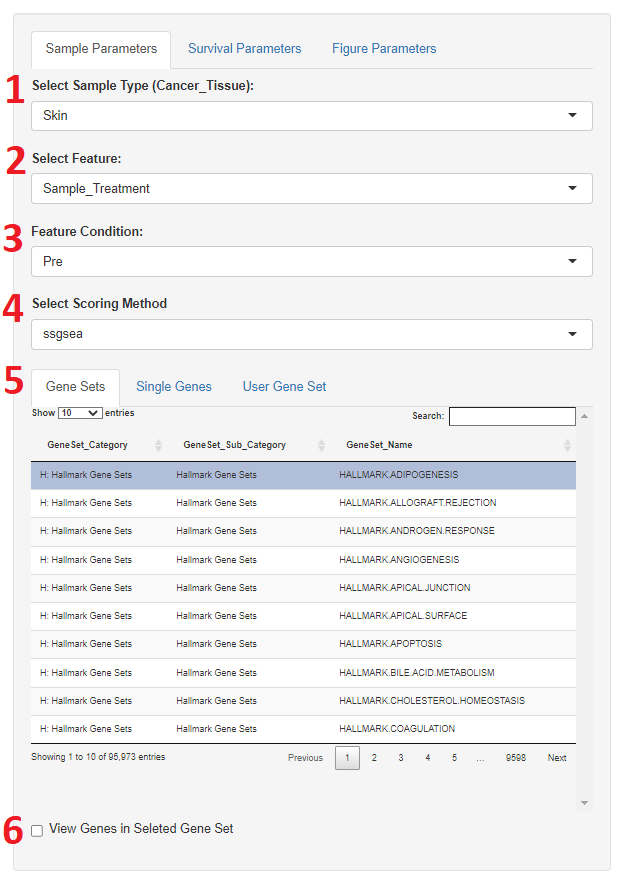

Supplement: Supplementary file 10 — Additional file 10. PATH-SURVEYOR-Suite-main.zip. [file 12859_2023_5393_MOESM10_ESM.zip › PATH-SURVEYOR-Suite-main/2-PATH-SURVEYOR-Interactive-App/App_Demo_Pictures/SideBar_SampleParameters.png]

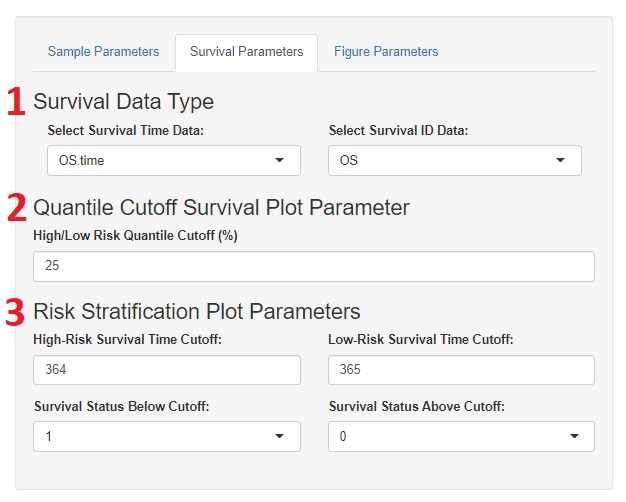

Supplement: Supplementary file 10 — Additional file 10. PATH-SURVEYOR-Suite-main.zip. [file 12859_2023_5393_MOESM10_ESM.zip › PATH-SURVEYOR-Suite-main/2-PATH-SURVEYOR-Interactive-App/App_Demo_Pictures/SideBar_SurvivalParameters.png]

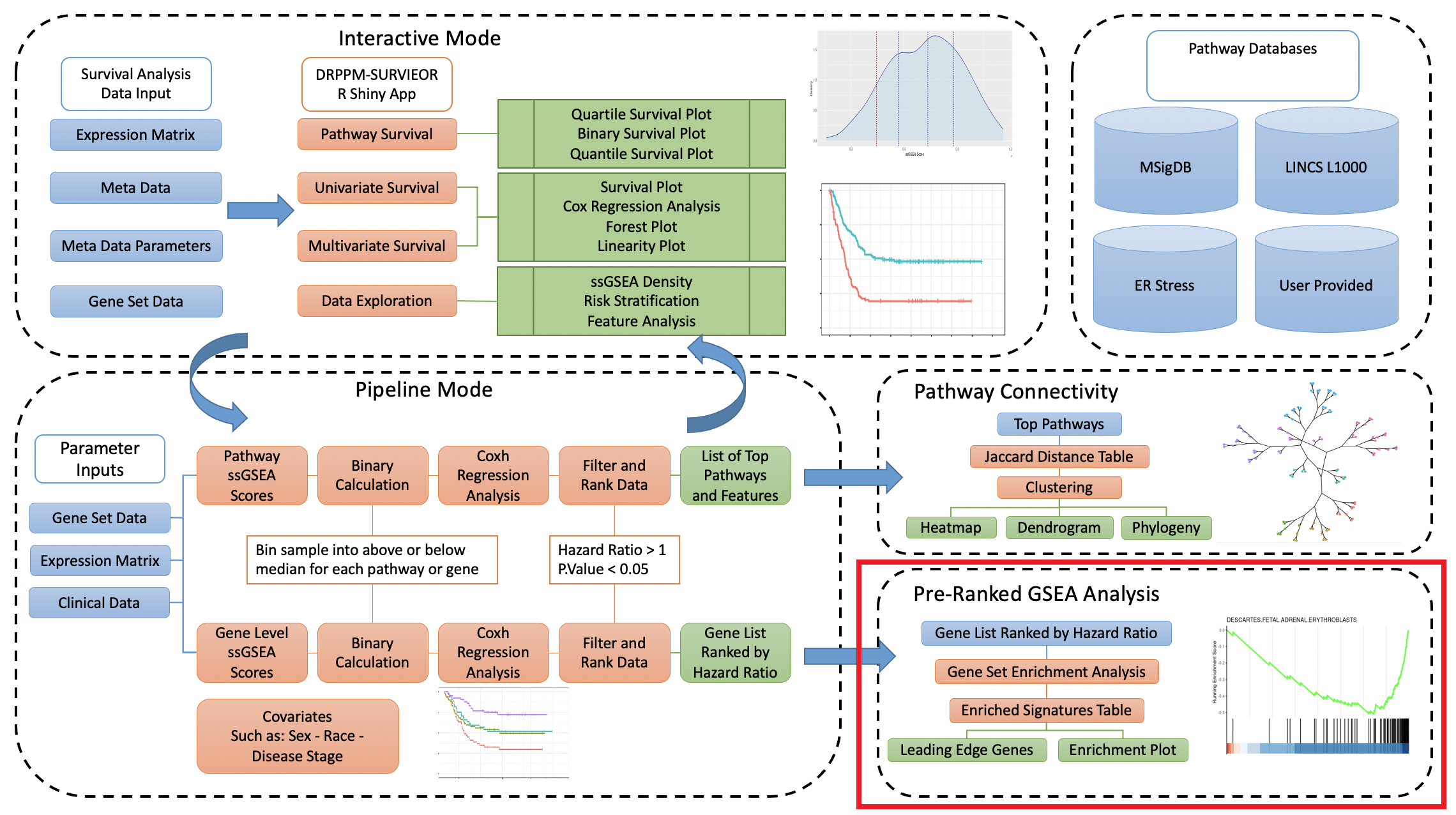

Supplement: Supplementary file 10 — Additional file 10. PATH-SURVEYOR-Suite-main.zip. [file 12859_2023_5393_MOESM10_ESM.zip › PATH-SURVEYOR-Suite-main/5-PreRanked-HazardRatio-GSEA-App/App_Pictures/FlowChart_PreRankedGSEA.png]

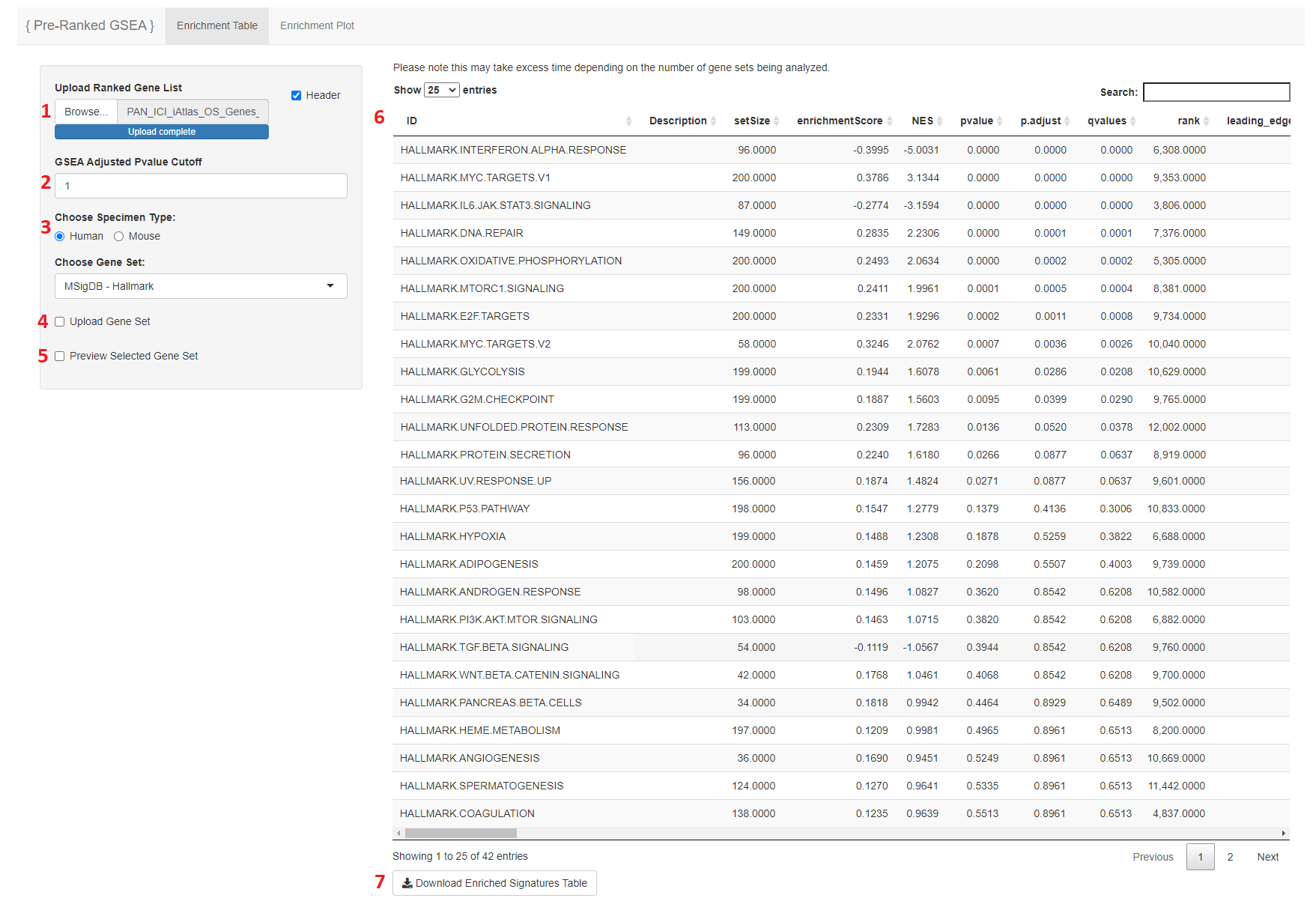

Supplement: Supplementary file 10 — Additional file 10. PATH-SURVEYOR-Suite-main.zip. [file 12859_2023_5393_MOESM10_ESM.zip › PATH-SURVEYOR-Suite-main/5-PreRanked-HazardRatio-GSEA-App/App_Pictures/PreRankGSEA_FirstTab.png]

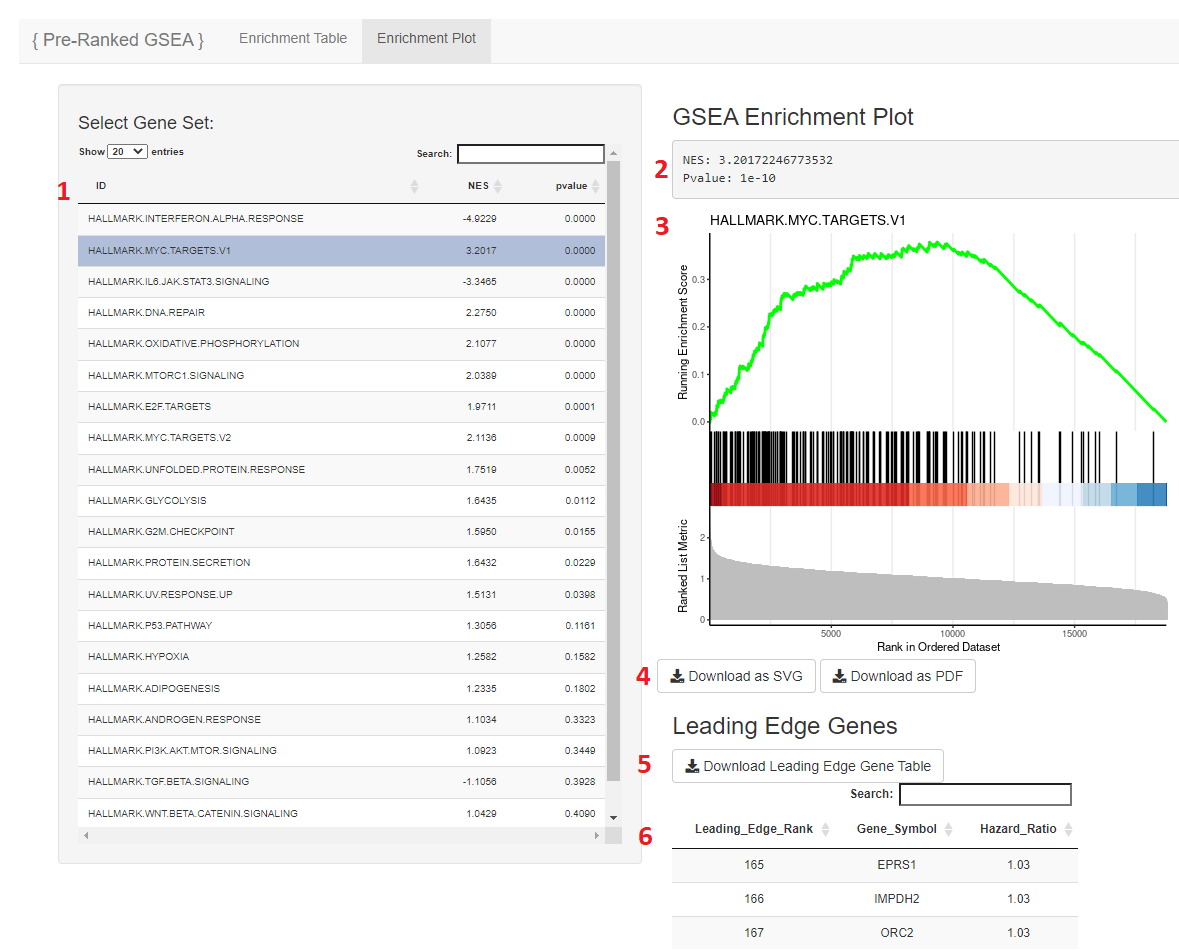

Supplement: Supplementary file 10 — Additional file 10. PATH-SURVEYOR-Suite-main.zip. [file 12859_2023_5393_MOESM10_ESM.zip › PATH-SURVEYOR-Suite-main/5-PreRanked-HazardRatio-GSEA-App/App_Pictures/PreRankGSEA_SecondTab.png]
